# Tailored Reaction Conditions and Automated Radiolabeling of [^177^Lu]Lu-PSMA-ALB-56 in a ^68^Ga Setting: The Critical Impact of Antioxidant Concentrations

**DOI:** 10.3390/ijms26199642

**Published:** 2025-10-02

**Authors:** Johanne Vanney, Léa Rubira, Jade Torchio, Cyril Fersing

**Affiliations:** 1 Nuclear Medicine Department, Institut Régional du Cancer de Montpellier (ICM), University Montpellier, 34090 Montpellier, France; 2 IBMM, University Montpellier, CNRS, ENSCM, 34293 Montpellier, France

**Keywords:** PSMA, ^177^Lu radiolabeling, anti-radiolysis, antioxidant: targeted radionuclide therapy, PSMA-ALB-56, albumin binder, automated synthesis, radiopharmacy

## Abstract

The growing use of experimental radiopharmaceuticals for targeted radionuclide therapy (TRT) highlights the need for robust “in house” radiolabeling protocols. Among these, PSMA-ALB-56 is a PSMA ligand incorporating an albumin-binding moiety to enhance pharmacokinetics, which showed promise for prostate cancer treatment. This study investigated manual radiolabeling conditions of this vector molecule with lutetium-177 and developed a corresponding automated synthesis protocol. Manual experiments on low activities explored buffer systems and antioxidants, identifying sodium acetate buffer and L-methionine as optimal, achieving radiochemical purities above 97% with excellent stability over 48 h. However, when these conditions were transposed directly to an automated process on a GAIA^®^ module with activities > 2 GBq, radiochemical purity dropped below 70% due to significant radiolysis. This result emphasized that conditions optimized at low activities are not directly transferable to high-activity automated production, and highlighted the crucial role of antioxidant concentration. An optimized automated method was subsequently developed, integrating a solid-phase extraction purification step, higher antioxidant levels during radiolabeling and formulation, and a larger final product volume. These changes led to radiochemical purities above 98.9% and excellent product stability over 120 h for 3 test batches. The presence of high concentrations of methionine and ascorbic acid was essential to protect against radiolysis. This work underscores the importance of adjusting radiolabeling strategies during process scale-up and confirmed that antioxidant concentration is essential for successful ^177^Lu radiolabeling. The optimized automated method developed here for [^177^Lu]Lu-PSMA-ALB-56 may also be adapted to other radiopharmaceuticals in development for TRT.

## 1. Introduction

The recent rise of targeted radionuclide therapy (TRT) has ushered in a new era in cancer treatment [1], offering precision and efficacy by delivering therapeutic radiation directly to cancer cells while minimizing the damage to healthy tissue [2]. Following the success of lutetium-177-radiolabeled somatostatin receptor ligands in clinical practice (e.g., [^177^Lu]Lu-oxodotreotide, LUTATHERA^®^, Novartis, Basel, Switzerland) [3], a rapidly expanding TRT approach now involves radiolabeled prostate-specific membrane antigen (PSMA) ligands [4]. This strategy became readily available with the regulatory approval of [^177^Lu]Lu-PSMA-617 ([^177^Lu]Lu-vipivotide tetraxetan, PLUVICTO^®^, Novartis, Basel, Switzerland) for the treatment of PSMA-positive metastatic castration-resistant prostate cancer (mCRPC) in patients previously treated with androgen receptor pathway inhibitors (ARPI) and taxane-based chemotherapy [5,6]. However, despite the excellent in vivo properties of such radiolabeled small molecule, the development of new PSMA ligands is still ongoing [7], especially to optimize their accumulation and retention in cancer tissues. In particular, one notable strategy to enhance the pharmacokinetics and therapeutic efficacy of TRT radiopharmaceuticals involves introducing a reversible albumin-binding moiety (ABM) into their chemical structure [8,9].

**Figure 1:**
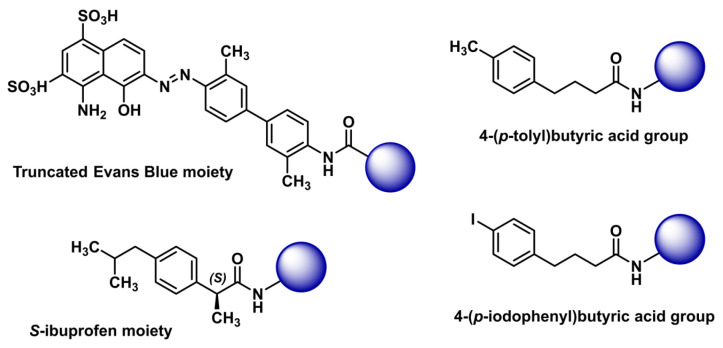
Main reversible albumin-binding moieties found in vector molecules for radioligand therapy. The blue sphere represents a vector molecule functionalized by ABM.

The principle of incorporating an ABM into TRT vector molecules is grounded in these moieties’ ability to bind reversibly to serum albumin, thus prolonging the blood circulation time of the radiopharmaceutical. This extended systemic presence facilitates greater tumor uptake and retention, thereby improving tumor-to-background absorbed dose ratios and enhancing therapeutic efficacy. In addition, the reversible binding ensures that the radiopharmaceutical can eventually dissociate from albumin [10,11], facilitating its uptake by target cancer cells. As a result, by extending the half-life of radiopharmaceuticals in the bloodstream, ABMs can improve bioavailability and tumor-to-background ratio [12,13,14]. Several chemical motifs have been used as ABMs, including a truncated variant of the Evans blue azo dye molecule, widely exemplified on both octreotide analogs [15,16,17,18,19,20,21,22] and PSMA ligands [23,24,25,26,27,28]. Ibuprofen has also recently been adopted as a low-affinity ABM [29,30,31,32,33]. Likewise, the 4-(*p*-iodophenyl)butyric acid and its derivative 4-(*p*-tolyl)butyric acid are among the most represented groups (Figure 1) [34,35]. These low molecular weight, reversible ABMs exhibit dissociation constants (*Kd*) with albumin in the low micromolar range, preventing excessive binding to the biomolecule and minimizing structural modifications to the initial molecular scaffold of the vector [34].

**Figure 2:**
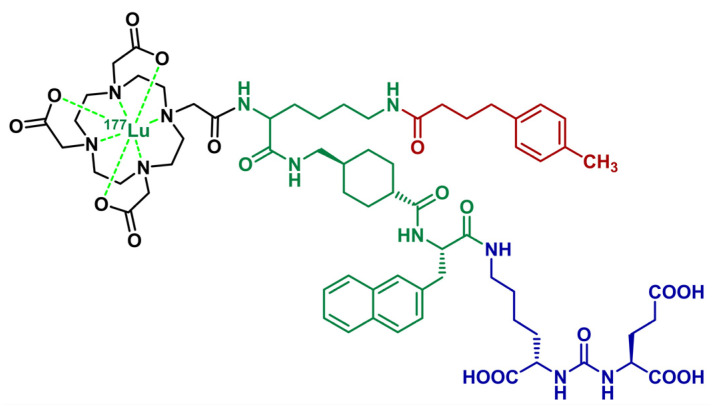
Chemical structure of [^177^Lu]Lu-PSMA-ALB-56, consisting of a PSMA-binding glutamate-urea-lysine unit (blue), a naphthylalanine/tranexamic acid/lysine spacer (green), a DOTA chelator (black) and a 4-(*p*-tolyl)butyric acid ABM (red).

Among the various series of PSMA ligands incorporating a 4-phenylbutanoic acid ABM [36,37,38,39,40,41], one has led to the identification of PSMA-ALB-56 [11]. This PSMA-617 derivative features an additional lysine between the DOTA chelator and the 2-naphthyl-L-alanine-tranexamic acid linker, functionalized by a 4-(*p*-tolyl)butyrate motif via an amide bond (Figure 2). [^177^Lu]Lu-PSMA-ALB-56 showed a 15-fold greater albumin binding than [^177^Lu]Lu-PSMA-617, with a comparable cell uptake internalization profile in vitro. Evaluation of [^177^Lu]Lu-PSMA-ALB-56 in 10 mCRPC patients (3.3 GBq, single dose) showed good tolerance and allowed reduction of baseline PSA values in 78% patients [42]. Normalized absorbed doses in tumor lesions were more than 2-fold higher than other therapeutic PSMA radioligands. Interestingly, doses to the salivary glands were similar; however, doses to the red marrow and the kidney were increased (~2.5-fold and ~3.5-fold, respectively). With regard to renal uptake, co-administration of cold PSMA ligands such as 2-(phosphonomethyl)pentanedioic acid (2-PMPA) to improve tumor-to-kidney ratio showed convincing results on a xenografted mouse model [43].

**Figure 3:**
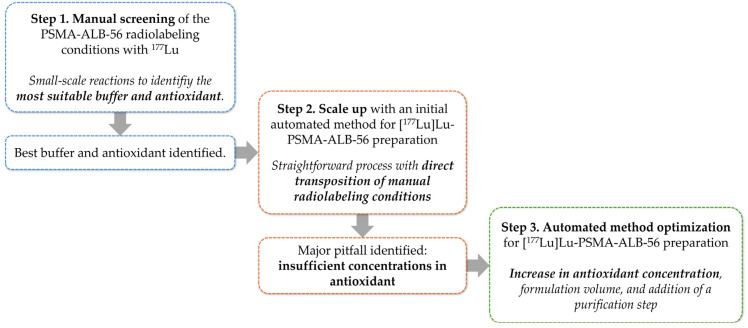
Schematic representation of the course of the present work.

As is often the case with TRT vectors, the ^177^Lu radiolabeling conditions applied to PSMA-ALB-56 in the literature are very standard and have not been extensively studied. More importantly, no automated preparation method for this TRT agent has been described in details, even though such processes have become a clinical standard in experimental radiopharmaceuticals production to ensure robustness, reproducibility, and low radiation exposure [44,45]. Therefore, the present work investigated the radiolabeling conditions of PSMA-ALB-56 with ^177^Lu through manual assays, specifically examining variations in the type and concentration of buffer solution, as well as the inclusion of antioxidant compounds in the reaction medium. Subsequently, the best conditions were transposed to two different automated synthesis processes (initial and optimized method, respectively) on the GAIA^®^ module to facilitate the clinical application of [^177^Lu]Lu-PSMA-ALB-56, aligning with standard requirements for experimental radiopharmaceutical preparation. Figure 3 shows the overall workflow. The concordance between manual and automated assays, particularly in terms of radiochemical purity (RCP), will be discussed.

## 2. Results and Discussions

### 2.1. Reaction Conditions Study at Small Scale

A total of 30 radiolabeling reactions, performed in 10 sets of triplicates (6 different buffers and 4 different antioxidants), were carried out manually. The radiolabeling conditions (95 °C heating for 15 min) were slightly adapted from the literature [11,46]. The total reaction volume was approximately 200 µL. The RCP of each reaction medium was determined exclusively by radio-HPLC, following established protocols, as reported in the literature [11]. Indeed, the conventional radio-TLC analysis conditions typically used for other PSMA ligands radiolabeled with gallium-68, such as [^68^Ga]Ga-PSMA-11, [^68^Ga]Ga-PSMA-617, or [^68^Ga]Ga-PSMA-I&T (i.e., ammonium acetate 1 M in a 1:1 methanol-water mixture [47]) do not effectively discriminate [^177^Lu]Lu-PSMA-ALB-56 from potential impurities, such as ^177^Lu^3+^ or [^177^Lu]Lu-DTPA.

#### 2.1.1. Influence of the Reaction Buffer

**Figure 4:**
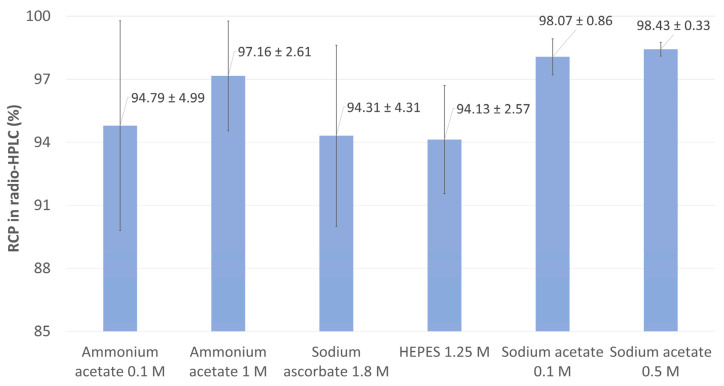
Mean RCP values measured by radio-HPLC when assessing the influence of reaction buffer on the radiolabeling of [^177^Lu]Lu-PSMA-ALB-56.

Six reaction buffers were tested (Figure 4), in line with ^177^Lu radiolabeling conditions reported in the literature. Adjusting the initial buffer pH to 4.5 maintained the medium at a reaction pH ideal for ^177^Lu radiolabeling [48]. It was not lowered by the addition of a minimal volume of 0.04 M HCl containing ^177^Lu. Interestingly, all tested conditions yielded very good to excellent RCP values in radio-HPLC, indicating that the reaction is somewhat permissive to buffers of varying nature and molarity. HEPES 1.25 M, known for its excellent results in ^68^Ga radiolabeling due to its low metallic complexation properties [49,50,51,52], resulted in the lowest RCP values (94.31 ± 2.57%). Sodium ascorbate 1.8 M, which can act both as a buffer and an antioxidant, produced comparable but slightly less consistent results (94.31 ± 4.31%). Similarly, ammonium acetate 0.1 M showed poorly reproducible RCP values (94.79 ± 4.99%). Notably, increasing the concentration of ammonium acetate by tenfold improved the mean RCP and reduced its standard deviation (97.16 ± 2.61%). The highest RCP values were achieved with sodium acetate, suggesting that this buffer is the most suitable for radiolabeling PSMA-ALB-56 with ^177^Lu, regardless of concentration. Indeed, the use of sodium acetate 0.1 M was associated with a mean RCP of 98.07 ± 0.86%. Increasing the buffer concentration to 0.5 M also allowed excellent RCP to be achieved (98.43 ± 0.33%), although not significantly higher than sodium acetate 0.1 M. In addition, the excellent suitability of sodium acetate for the preparation of [^177^Lu]Lu-PSMA-ALB-56 demonstrated here aligns with reaction conditions reported in the literature, which mention the use of sodium acetate 0.5 M [11,36]. Consequently, sodium acetate 0.1 M was selected for subsequent assays, as its low concentration was preferred for precise control of reaction pH without the need for adjustment with HCl 37% [53]. Of note, although increasing some buffers concentration has been shown to reduce radioconjugate degradation [54], this effect becomes negligible at activity levels corresponding to a patient dose. The additional use of appropriate quenchers will therefore remain necessary.

#### 2.1.2. Influence of Antioxidant Agents

**Figure 5:**
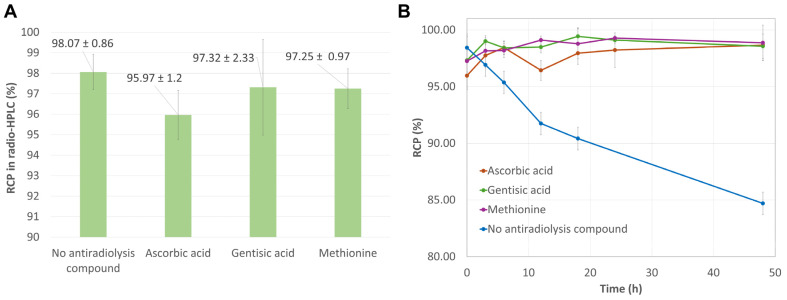
(**A**) Mean RCP values measured by radio-HPLC at EoS when assessing the influence of an ARC on the radiolabeling of [^177^Lu]Lu-PSMA-ALB-56; (**B**) Time course of the RCP of [^177^Lu]Lu-PSMA-ALB-56 measured by radio-HPLC, in the presence of ARCs, for low activity samples. Some RCP values may appear to increase over time due to signal integration uncertainties related to background noise.

The importance of adding an antioxidant compound to the final formulation of [^177^Lu]Lu-PSMA-ALB-56 was demonstrated by Umbricht et al., who showed that although the radioligand remained stable for 4 h at room temperature (RCP > 93% at a 0.5 MBq/µL radioactive concentration), it degraded due to radiolysis after 24 h of incubation, whereas a RCP > 96% could be maintained for at least 24 h in the presence of *L*-ascorbic acid [11]. Indeed, the beta-minus decay of ^177^Lu (E_max_ ß- = 498 keV [79.3%] [55]) can lead to either direct damage to the vector molecule from the resulting beta-minus particles, or primarily to degradation of the vector molecule due to its interaction with radicals formed by water radiolysis. In our study, the addition of 10 mM cysteine to the reaction medium completely inhibited the radioelement complexation, resulting in 100% free ^177^Lu. One possible explanation for these results could be that cysteine, via its thiol group capable of binding metal ions, competes with the DOTA chelator and strongly disrupts the formation of [^177^Lu]Lu-PSMA-ALB-56 [56]. The results obtained with this quencher are inconsistent with those of Larenkov et al., who demonstrated the value of using a mixture of 7.4 mM cysteine and DMSA as stabilizers during the synthesis of [^177^Lu]Lu-PSMA-617 [53]. Conversely, a concentration of 3.5 mM ascorbic acid in the reaction medium led to an average RCP of 95.97 ± 1.2% at EoS. The radiolabeled PSMA ligand showed excellent stability over 48 h, with average RCP values exceeding 96% over this period (Figure 5). In comparison, the RCP of [^177^Lu]Lu-PSMA-ALB-56 radiolabeled without any AR compound tended to decrease over time, dropping around 85% after 48 h. The addition of either gentisic acid at 3.5 mM or methionine at 10 mM resulted in excellent RCP at EoS (97.32 ± 2.33% and 97.25 ± 0.97%, respectively), with mean RCP greater than 97% over 48 h. Gentisic acid, which exerts its antioxidant action by converting to the quinone form 2,5-dioxobenzoic acid [57], is widely used in radiopharmaceutical formulations [58]. For example, up to 6 µg per vial can be found in cold kit formulations of somatostatin analogs for ^68^Ga radiolabeling, such as SomaKit TOC^®^ (Novartis, Basel, Switzerland) [59] and NetSpot^®^ (Novartis, Basel, Switzerland) [60]. On the other hand, methionine is not currently used in commercial radiopharmaceutical formulations but is frequently reported in radiolabeling protocols for experimental compounds, most often involving ^68^Ga-labeled vectors and, more rarely, ^177^Lu-labeled molecules. For instance, the team of de Blois identified 3.5 mM methionine as an excellent quencher for [^177^Lu]Lu-PSMA-617 (RCP > 95% at 24 h when combined with 10% ethanol) in a study involving small-scale radiolabeling conditions very similar to ours (80 MBq ^177^Lu, 140 µL reaction volume, ~42 MBq/nmol molar activity) [61]. It is relevant to mention that methionine can be combined with other antioxidants, such as ascorbic acid in the automated preparation protocol of ^68^Ga/^177^Lu-labeled FAP-2286 reported by Baum et al. [62]. Moreover, some radiolabeling protocols even combine methionine with 2 or 3 other anti-radiolysis agents in the reaction medium. This is especially useful when radiolabeling peptide vectors that contain an oxidation-sensitive methionine residue, such as the cholecystokinin-2 receptor (CCK-2) agonist [^68^Ga]Ga-DOTA-CP04 (using ethanol, sodium ascorbate, gentisic acid and methionine) [63] or the CCK-2-targeting peptide [^111^In]In-DOTA-MG11 (using ethanol, ascorbic acid and methionine) [64]. Of note, selenomethionine could also exhibit interesting scavenging properties in ^177^Lu radiolabeling reactions [65], although its translation to clinical use may be more complex due to regulatory constraints. Based on the same rationale, the anti-radiolytic efficacy of both gentisic acid and methionine has also been demonstrated, when used separately or in combination, in the ^177^Lu radiolabeling of DOTA-H2MG11, a particularly radiosensitive minigastrin analog [66]. Interestingly, this effect appeared to be pH-dependent and was maximal at pH 5.0–5.5, maintaining relative stability of the preparation for almost 7 days (RCP > 90% at EoS to RCP > 80% at 6.9 d). Overall, given the supporting literature alongside the excellent RCP of [^177^Lu]Lu-PSMA-ALB-56 and the very good stability of the radiocomplex over at least 48 h in the presence of 1.45 mg/mL methionine (reaction medium concentration), this amino acid has been identified as the AR compound of choice.

**Figure 6:**
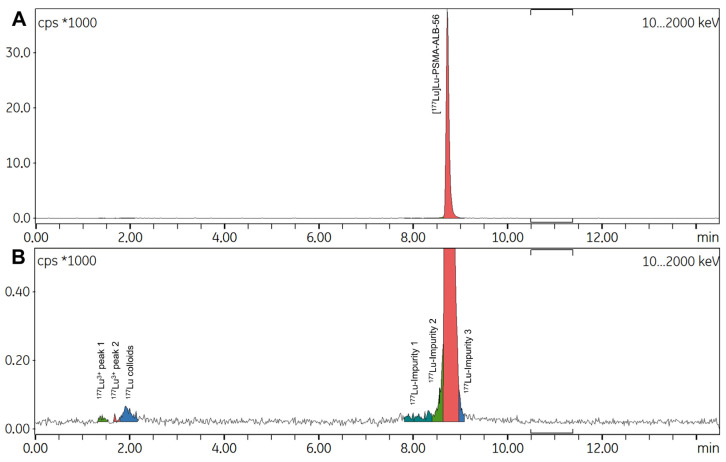
(**A**) Representative radio-HPLC spectrum of [^177^Lu]Lu-PSMA-ALB-56 obtained with manual radiolabeling under the best reaction conditions described above and with low ^177^Lu activity (~74 MBq). (**B**) Zoomed-in view of the same spectrum focusing on the baseline and showing absence of radiolysis by-products.

In summary, the optimal manual radiolabeling conditions for preparing [^177^Lu]Lu-PSMA-ALB-56 consisted of 160 µL of sodium acetate buffer 0.1 M, 10 µL of L-methionine 30 mg/mL, and 30 µg of vector, combined with approximately 2 mCi of ^177^Lu in 0.04 M HCl (7–8 µL). Heating this mixture at 95 °C for 15 min yielded a product with high RCP and stability (>97% over 48 h) without the need for further purification (Figure 6). These conditions were subsequently scaled up and adapted to a first automated synthesis protocol.

### 2.2. Design of the Initial Automated Radiolabeling Protocol and First Preparation of High-Activity Doses

#### 2.2.1. Conception of the Automated Procedure

An initial automated sequence for the synthesis of [^177^Lu]Lu-PSMA-ALB-56 was set up on the GAIA^®^ module, using the best reaction conditions previously identified. Automated preparation protocols for ^177^Lu-labeled radiopharmaceuticals often involve the addition of all reagents into the vial containing the radioisotope solution, which is then heated. In our case, to avoid using the original ^177^Lu vial format which may not be compatible with all heating block models, the protocol was adapted to transfer lutetium-177 chloride directly to the captive reaction vial within the module’s tubing set. To limit the loss of radioactivity due to remnants of [^177^Lu]LuCl_3_ in its initial vial, a first dilution step with the buffered PSMA-ALB-56 solution was implemented to facilitate radioactivity transfer to the reaction vial. A subsequent rinsing step of the ^177^Lu vial with HCl 3.6 mM ensured improved recovery of the radioisotope in the reaction vial. Closely related procedures have been described in the literature, involving either rinsing with the vector solution [67] or pre-dilution of [^177^Lu]LuCl_3_ with ultrapure water [68]. Given the excellent RCP values obtained in the manual tests, final purification of the peptide using an SPE cartridge did not appear necessary. Nevertheless, DTPA was added after radiosynthesis to chelate residual ^177^Lu^3+^, particularly to prevent its in vivo accumulation in bone [48,69]. According to literature, DTPA can be added to a radiopharmaceutical formulation to complex free radionuclides and allow fast renal excretion [70]. Alternatively, the simple addition of a CM cartridge (pre-conditioned with 10 mL of WFI) on top of the final vial could also be considered, as it would not extend the preparation time while retaining unreacted ^177^Lu^3+^ without requiring elution. However, this approach is more commonly employed in ^68^Ga-radiolabeling protocols and may also retain a portion of the radiolabeled product [71].

#### 2.2.2. Production of Test Batches

**Figure 7:**
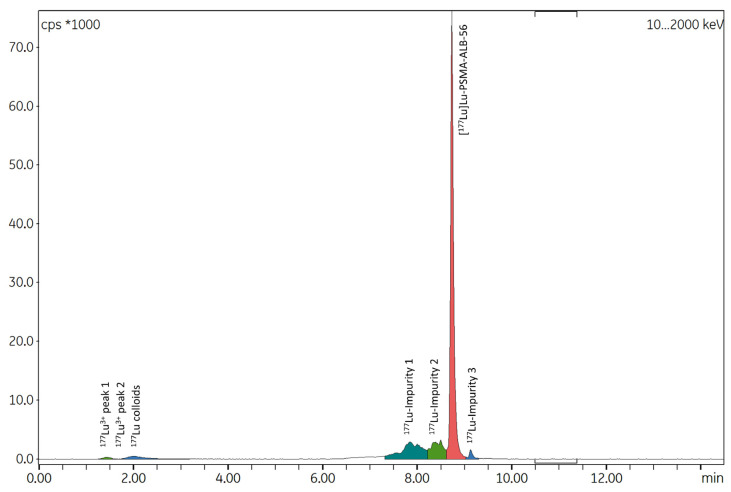
Representative radio-HPLC spectrum of [^177^Lu]Lu-PSMA-ALB-56 prepared using initial automated process (without SPE) under the best reaction conditions described above and with high ^177^Lu activity. RCP = 68.11%.

Three automated test syntheses were performed using this initial protocol, with mean starting activities of ^177^Lu at 2789 ± 153 MBq and 65 µg of vector. At the end of the preparations, 86.22 ± 6.1% of the initial radioactivity was recovered in the final vial, indicating minimal losses within the tubing set. Notably, 10.72 ± 5.63% of the initial activity remained in the original ^177^Lu vial despite two rinsing steps. Although this automated protocol was functional, radio-HPLC analysis of the radiolabeled products revealed, in all three syntheses, a significant proportion of radiolysis by-products immediately at EoS, resulting in RCP below 70% (Figure 7). In contrast, the proportion of free ^177^Lu^3+^ and ^177^Lu colloids consistently remained below 3%.

#### 2.2.3. Possible Causes for the Formation of Radiolysis Byproducts

The formation of side-products, including during the radiolabeling reaction course, is a well-known phenomenon with both beta-minus [72] and beta-plus emitters [73], often reported as activity-dependent. Several parameters may contribute to the formation of radioimpurities. In the present case, a low RCP was observed directly at EoS, indicating a potential issue during the radiolabeling process. Among the possible contributing factors to the formation of radioimpurities, molar activity can be considered. Indeed, a high molar activity (i.e., the amount of radioactivity per mole of vector molecule) can enhance radiolabeling yields, but it may also increase radiolysis and reduce the stability of the radiolabeled product [61]. The average molar activity during the manual tests presented was only around 3.3 MBq/nmol. For the automated preparations described here, this parameter was nearly 15 times higher, reaching 49.3 ± 6 MBq/nmol. This value is comparable to the highest molar activities reported in the literature for PSMA-ALB-56 used in vitro and in preclinical studies [11]. For clinical applications, the highest molar activity of [^177^Lu]Lu-PSMA-ALB-56 was approximately 42 MBq/nmol [42]. Increasing the amount of vector used in the reaction could therefore be a strategy to improve the overall outcome of the radiolabeling step. However, the preparation of other ^177^Lu-labeled PSMA ligands is frequently reported with much higher molar activities. For instance, one study describes the manual production of [^177^Lu]Lu-PSMA-I&T with a specific activity of 58 MBq/nmol [74]. Automated protocols for the preparation of the same radiopharmaceutical achieve even higher molar activities, reaching 129 MBq/nmol [71] or even 225 MBq/nmol, with stability maintained for more than 7 days [68]. Another related vector molecule, PSMA-617, can be radiolabeled in-house with ^177^Lu to achieve molar activities around 90 MBq/nmol [67]. This parameter is therefore unlikely to be the main contributing factor.

Reaction temperature and heating time are critical parameters to consider, as they may impact the stability of both the radiocomplex and the PSMA ligand. An insightful study by Martin et al. revealed that the Glu-CO-Lys motif used in PSMA-targeted radiotracers can undergo spontaneous cyclization to form stable five-membered rings [75]. This side reaction likely affects other similar tracers and was shown to be temperature-dependent. For clinical production, labeling was performed in the same study at 75 °C for 45 min in 500 μL of ammonium acetate buffer, yielding [^177^Lu]Lu-PSMA-617 with ≥95% RCP at activities up to 16 GBq. However, additional radiolysis byproducts still appeared for activities ≥ 8 GBq. In the literature, [^177^Lu]Lu-PSMA-ALB-56 is typically prepared by heating at 95 °C for 15 min, under conditions identical to those used in our study [11,36]. Moreover, automated radiolabeling protocols for other PSMA ligands report similar or even more stringent conditions [67], such as longer heating times (e.g., 30 min instead of 15 min [74]) or higher temperatures (e.g., 105 °C instead of 95 °C [68]).

The reaction and final preparation volumes should also be taken into account, as they may lead to a self-quenching effect if sufficiently large [76]. In a comprehensive study on the causes of radiolysis during the preparation of [^177^Lu]Lu-PSMA-617, Larenkov et al. demonstrated that the RCP was inversely proportional to the absorbed dose in the preparation, which in turn was directly related to the volume activity [53]. In our case, a reaction volume of 3 mL was used, which is generally consistent with the literature on automated ^177^Lu radiolabeling of other PSMA ligands [68,71]. Some studies have reported even lower reaction volumes of 2 mL or even 1.5 mL [67,74], despite reaching higher molar activities than in our study, around 90 and 60 MBq/nmol, respectively. In contrast, our final preparation volume was approximately 10 mL, which is lower than that reported in most other studies, where final volumes for ^177^Lu-labeled preparations are typically around 20 mL [68,74,77,78]. The benefit of a larger final volume on the radiochemical stability of [^177^Lu]Lu-PSMA-I&T preparations has also been demonstrated [79]. Therefore, a slight increase in the reaction volume, along with immediate post-synthesis dilution using 0.9% NaCl, could be considered to help reduce the formation of radiolytic side-products [71,80].

Ultimately, the most determinant factor affecting the course of the automated radiolabeling of [^177^Lu]Lu-PSMA-ALB-56 presented here is very likely the amount of antioxidant used, both in the reaction mixture and in the final formulation. In this study, the quantities selected were sufficient to exert a significant quenching effect during low-activity radiolabeling, but proved markedly insufficient under scaled-up conditions. By comparison, the amounts used in the initial automated trials (i.e., 5 mM methionine in the reaction medium) are more consistent with concentrations typically reported in ^68^Ga radiolabeling protocols [62,63]. Indeed, quenchers, either alone or in combination, are routinely used in high quantities in radiolabeling reactions involving beta emitters [72]. For instance, the literature reports the use of gentisic acid at a concentration of 25 mg/mL (around 160 mM) for the automated synthesis of [^177^Lu]Lu-3BP-3940 [81]. The same concentration is found in the formulation of freeze-dried cold kits for [^177^Lu]Lu-PSMA-617 preparation [82], and slightly lower amounts (around 20 mg/mL) have been used for the production of the vitronectin receptor antagonist [^90^Y]Y-RP697 [83]. Similar levels of antioxidants are also used post-reaction to maintain sufficient stability over time. Surprisingly, this parameter is rarely investigated in radiolabeling automation studies, likely because the doses produced “in-house” are generally administered shortly after synthesis, once release quality control tests are completed. Among the anti-radiolysis compounds, ascorbate holds a particular place [54]. It is frequently employed and even commercially available in reagent sets for ^177^Lu radiolabeling (e.g., by Polatom or ITM). It is also relevant that implementing a final SPE step requires subsequent readdition of quencher to preserve stability, as demonstrated by Maus et al., who reported sustained RCP > 95% for [^177^Lu]Lu-DOTATATE over 72 h with the addition of 100 mM ascorbic acid in a final volume of 5 mL post-SPE [84]. Interestingly, this study also showed that diluting the quencher in 20 mL instead of 5 mL reduced its effectiveness, indicating that the concentration of the anti-radiolysis agent is more critical than the dilution effect of the final volume. Finally, it is also relevant that ethanol used in the SPE cartridge elution can contribute to quenching because of its antioxidant properties [72,85].

### 2.3. Design of the Optimized Automated Radiolabeling Protocol and Second Preparation of High-Activity Doses

#### 2.3.1. Modifications to the Initial Automated Procedure

Based on the previous findings, a new automated method for [^177^Lu]Lu-PSMA-ALB-56 preparation was designed to optimize the stability of the radiocomplex. Specifically, the aim was to minimize the formation of degradation byproducts by: (i) significantly increasing the amount of antioxidant (both in the reaction mixture and the final formulation) to align more closely with protocols described in the literature; (ii) implementing a final SPE step to purify the crude reaction mixture and benefit from the radioprotective properties of a formulation containing 10% ethanol; (iii) doubling the final product volume to 20 mL to potentially enhance radiochemical stability through a self-attenuation effect.

Methionine was reused as a quencher in the reaction mixture, but its concentration was increased to 18.25 mg/mL (around 123 mM, approximately 25 times higher than in the initial method) to ensure its effectiveness. To maintain the stability of the radiopharmaceutical in its final formulation, 0.5 mL of ascorbic acid 50 mg/mL plus sodium ascorbate 100 mg/mL was manually injected into the final vial through the 0.22 µm filter before the start of synthesis, resulting in a concentration of 3.6 mg/mL at EoS. The acetate buffer was kept unchanged, with a reaction volume of approximately 2.5 mL. The DTPA solution, previously added during and after radiolabeling, was replaced by the SPE cartridge elution solutions, consisting of 17.2 mL of NaCl 0.9% and 3 mL of ethanol 60% in WFI, resulting in a final vial volume of approximately 20.7 mL. The SPE cartridge was selected with a sufficiently large sorbent volume (360 mg) to allow easy fluid flow and avoid any reduction in flow rate through the cartridge. The overall synthesis time was not increased by the addition of this purification step; on the contrary, it decreased slightly from 28.25 min with the initial method to 28.1 min with the optimized method. Although this total duration may appear long compared to a conventional ^68^Ga synthesis, it does not significantly impact the final activity of the radiopharmaceutical given the long half-life of lutetium-177.

#### 2.3.2. Production of Test Batches

**Figure 8:**
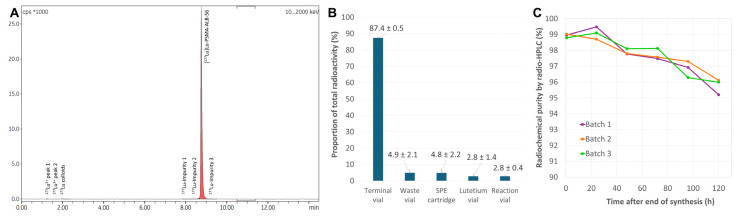
(**A**) Representative radio-HPLC spectrum of [^177^Lu]Lu-PSMA-ALB-56 prepared by optimized automated process, with high ^177^Lu activity; (**B**) Radioactivity distribution in the cassette at the end of an automated synthesis; (**C**) Time course of the RCP of [^177^Lu]Lu-PSMA-ALB-56 measured by HPLC, in the presence of ascorbic acid 1.2 mg/mL and ascorbate 2.4 mg/mL (final concentrations), for high activity samples (n = 3; mean activity at EoS = 1981 ± 110 MBq). Some RCP values may appear to increase over time due to signal integration uncertainties related to background noise.

Three test batches were then produced using this method. Unlike the preparations obtained with the initial protocol, the RCP of these radiolabeling products was excellent at EoS (mean value by radio-HPLC: 98.92 ± 0.12%) with no significant traces of radiolysis by-products (Figure 8A). It is important to note that this process also provided very good RCYs (mean value adjusted by RCP determined by HPLC: 86.52 ± 0.48%). Less than 5% of the activity engaged in the reaction was found in the waste vial and on the C_18_ cartridge at EoS, indicating both an efficient radiolabeling and an easy elution from the SPE cartridge. Less than 3% of the activity remained in the commercial ^177^Lu vial and in the reaction vial (Figure 8B). The effectiveness of high concentrations of ascorbic acid/ascorbate in the final formulation was evaluated by measuring the RCP of the three test batches over time. Measurements performed between EoS and 120 h post-EoS all yielded RCP values greater than 95%, demonstrating the excellent stability of [^177^Lu]Lu-PSMA-ALB-56 under these conditions (Figure 8C). Nevertheless, it is important to mention that the average activity engaged in these test syntheses was 2460 ± 103 MBq, resulting in final preparations with a mean activity of 1981 ± 110 MBq. Therefore, initial activities approximately 60% higher would be needed to produce patient doses of usual activity (i.e., 3.3 GBq) [42].

Additional quality controls were carried out on these 3 test batches, similarly to selected analyses performed on validation batches in the context of investigational medicinal product dossiers. Results are summarized in Table 1. Specifically, the radionuclide used met the identity and radionuclide purity criteria expected for ^177^Lu n.c.a.-grade. The pH of the radiolabeled products was measured at 4.5 for each batch, in line with the high amounts of ascorbic acid present in the final formulation. This pH remains compatible with intravenous administration [86,87,88], although a slow infusion rate (e.g., 1 mL/min) would be recommended to minimize the risk of irritation of peripheral veins. As previously mentioned, RCP at EoS was excellent and close to 99% for the 3 batches. For the activities used, the radiocomplex remained stable for at least 120 h, opening the possibility for doses preparation several days prior to use. By engaging 65 µg of vector in the reactions, the molar activities and specific activities of the test batches reached average values of 40.12 ± 2.28 GBq/µmol and 30.16 ± 1.71 MBq/µg, respectively.

The automated method described here was exemplified using PSMA-ALB-56; however, from a functional perspective, this synthesis protocol could also be adapted for the ^177^Lu radiolabeling of other PSMA ligands incorporating an ABM, such as Ludotadipep recently under clinical evaluation [89], SibuDAB [90], or the latest P17-088 derivative [41].

## 3. Materials and Methods

### 3.1. Chemicals, Solvents and Equipment

#### General Information

Radiolabeling assays were conducted on non-GMP grade PSMA-ALB-56 (MedChem Express, Monmouth Junction, NJ, USA) with a purity > 98.9%. An initial stock solution of PSMA-ALB-56 (1 mg/mL in WFI) was aliquoted into LoBind tubes as 30 µg and 65 µg fractions and stored at −20 °C for up to 3 months. All chemicals used for the radiolabeling reactions were of the highest purity grade and were sourced from Merck (Darmstadt, Germany). All reagents were used without further purification. Pharmaceutical-grade WFI (Eau pour prép. injectables 10 mL PROAMP^®^, Aguettan, Lyon, France) was used to prepare the various solutions used for radiolabeling assays. Lutetium-177 non-carrier added (n.c.a.) was obtained from ITG (Isotope Technologies Garching GmBH, Garching, Germany) as radiochemical grade [^177^Lu]LuCl_3_ in 0.04 M HCl. The manual study of radiolabeling conditions was carried out on a batch of 10 GBq/mL ^177^Lu in 210 µL with a specific activity of 3.268 GBq/mg at activity reference time (ART). A first series of [^177^Lu]Lu-PSMA-ALB-56 automated productions (initial method; n = 3) used vials of 2.6−2.9 GBq ^177^Lu in 530 µL with a specific activity of 2.536 GBq/mg at ART. A second series of [^177^Lu]Lu-PSMA-ALB-56 automated productions (optimized method; n = 3) used vials of 2.3−2.6 GBq ^177^Lu in 100 µL with a specific activity of 3.191 GBq/mg at ART. Radiolabeling assays and quality controls took place in a radiopharmaceutical preparation unit (GMP grade C cleanroom).

### 3.2. Radiolabeling Conditions Study

Manual radiolabeling assays for the reaction conditions study were conducted in a Medi2000 Iode^®^ shielded cell (LemerPax, La Chapelle-sur-Erdre, France). Prior to reactions, the buffer solutions and the anti-radiolysis (AR) compound solutions to be tested were prepared extemporaneously in sterile glass vials (TC-ELU-5^®^, Curium, Paris, France) and using plastic spatulas to weigh the powders, taking care to avoid metallic contamination. A calibrated precision balance (320XB, Precisa Gravimetrics, Dietikon, Switzerland) was used to weigh in the correct buffer amounts and calibrated precision micropipettes (Pipetman^®^, Gilson, Middleton, WI, USA) with sterile disposable tips were used for all liquids transfers. Particular care was taken to adjust the pH of the buffer solutions to 4.5 using 37% HCl. The effective pH was checked on an aliquot of each buffer solution using a recently calibrated Vario^®^ pH meter (WTW^®^, Xylem, Washington, DC, USA) equipped with a SenTix^®^ 41 pH electrode (WTW^®^, Xylem, USA). For the study of reaction conditions, a single parameter (type or concentration of buffer, presence and type of AR compound) was modulated at a time. The buffers and AR compounds tested were chosen based on ^177^Lu radiolabeling conditions described in the literature. Table 2 summarizes the bibliographic sources for the tested conditions. All conditions were tested in triplicate.

Standard manual radiolabeling was carried out as follows: in a 1.5 mL Eppendorf tube containing 30 µg PSMA-ALB-56 in 30 µL WFI were added 160 µL of appropriate buffer solution, 7.4 µL of ^177^Lu (~74 MBq) and 10 µL of AR compound solution (if needed). The reaction medium was gently mixed by repeatedly pipetting the solution up and down 3 times, then the Eppendorf was capped and placed during 30 min in a water bath set at 95 °C. The effective bath temperature was monitored by an external thermometer. At the end of preparation and before performing quality controls, 200 µL of a 4 mg/mL DTPA solution were added to the reaction medium [69,84], then the Eppendorf was left to cool for 15 min.

### 3.3. Initial Automated Radiolabeling Protocol Without Terminal Purification

The automated radiosynthesis of [^177^Lu]Lu-PSMA-ALB-56 was developed and performed on a GAIA^®^ synthesis module (Elysia-Raytest, Straubenhardt, Germany) operated by the GAIA control software version 2.2 (Elysia-Raytest, Straubenhardt, Germany) and using sterile, single use cassettes for synthesis (Fluidic kit RT-01-H ABX, Advanced Biochemical Compounds, Radeberg, Germany). Syntheses were conducted in a GMP grade A shielded cell with laminar airflow (MEDI 9000 Research 4R, LemerPax, La Chapelle-sur-Erdre, France), which housed the automated synthesis module. The radioactivity in product vials was measured with a calibrated ionization chamber (CRC^®^-25R, Capintec, Florham Park, NJ, USA).

**Figure 9:**
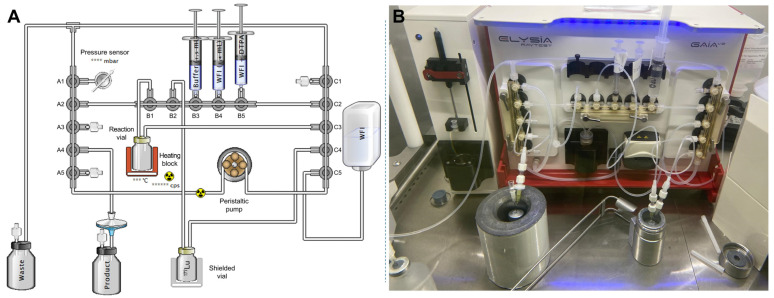
(**A**) Software system for the automated preparation of [^177^Lu]Lu-PSMA-ALB-56 with the GAIA^®^ synthesizer according to initial method; (**B**) Cassette setup on the GAIA^®^ module for the synthesis of [^177^Lu]Lu-PSMA-ALB-56 according to initial method.

Prior to synthesis start, a disposable cassette was installed on the synthesis module as illustrated in Figure 9. The GAIA^®^ module features 3 manifolds (A, B and C from left to right on the module) of five 3-way valves each (1 to 5, from top to bottom for manifold A and C, and from left to right for manifold B), a heating block designed to hold the reaction vial, and a peristaltic pump for transferring liquids throughout the synthesis process. Subsequently, reagents were prepared and connected to the kit. In particular, the PSMA-ALB-56 vector (65 µg in 65 µL WFI for one automated radiolabeling) was mixed with 1.425 mL of appropriate buffer solution supplemented with 75 µL of methionine 30 mg/mL, transferred to a 3 mL syringe and connected at B3 (manifold B, valve 3) position. Then, 1.4 mL of HCl 3.6 mM pH 4.5 (i.e., 50 µL of HCl 0.1 N in 1.35 mL WFI) were conditioned in a 3 mL syringe and connected at B4 as a rinsing solution for the ^177^Lu vial. Likewise, 7 mL of DTPA 1 mg/mL were prepared in a 10 mL syringe and connected at B5 for adjunction in the reaction medium after radiolabeling. Of note, all liquid transfers into syringes were carried out using Sterican^®^ needles (B. Braun, Melsungen, Germany) with a silicone coating on the inner wall to prevent contamination by metal traces from the needle’s chromium-nickel stainless steel. A bottle of WFI (Ecoflac^®^, B. Braun, Melsungen, Germany) was also connected at C5 for tubing rinsing operations during the automated sequence. Finally, the vial containing ^177^Lu was positioned in front of the synthesizer in a shielded container equipped with a pierced lead cap. Two lines were then connected to its septum: the first from C4, terminated by a short needle and serving as an air inlet, and the second from B2, terminated by a long needle dipping to the bottom of the vial and used to withdraw the liquid from the vessel.

Automated synthesis proceeded as follows: first, a kit integrity test was carried out to check for any leaks in the system and avoid any spillage during synthesis. To this end, filtered air was pumped into the system to raise the pressure in the tubing to 1500 mbar. The system was then closed, and if the observed pressure drop was no greater than 400 mbar over 20 s, integrity was confirmed and the synthesis proceeded. The initial step involved adding the buffer solution containing PSMA-ALB-56 to the ^177^Lu vial to dilute the radioisotope solution and facilitate precursor transfer to the reaction vial. The lutetium vial was then rinsed with 1.4 mL of HCl 3.6 mM from the syringe at B4, which was also transferred to the reaction vial before purging the lines with filtered air. Heating, initiated at a setpoint temperature of 60 °C in the previous step, was pursued at 95 °C for a total duration of 15 min after addition of ^177^Lu. While radiolabeling was in progress, the manifolds of the kit were rinsed with WFI and purged with filtered air, particularly manifold B, through which free ^177^Lu flowed in the B1–B2 section. To complete radiolabeling, a first 3 mL portion of 1 mg/mL DTPA solution was added to the reaction medium, which was then transferred to the product vial. The remaining DTPA solution (~4 mL) was used to rinse the reaction vial and was subsequently transferred to the product vial. Sterilizing filtration was achieved with a 0.22 μm end filter, and the filter’s integrity was verified by the synthesizer at the end of the preparation via a bubble point test, with minimum pressure value set at 2.5 bar. At the end of the synthesis (EoS), the reaction yield was calculated to estimate the proportion of radioactivity actually recovered in the terminal vial at the end of preparation. This was calculated by comparing the activity collected in the product vial with the sum of the post-synthesis activities in the initial ^177^Lu vial, reaction vial, waste vial and product vial. Radiochemical yield (RCY) was calculated by multiplying the reaction yield by the RCP. The complete [^177^Lu]Lu-PSMA-ALB-56 radiosynthesis procedure on the GAIA^®^ module is outlined in Table 3, with detailed automated sequence available as Supplementary Materials Data.

### 3.4. Optimized Automated Radiolabeling Protocol with Terminal Sold-Phase Extraction

**Figure 10:**
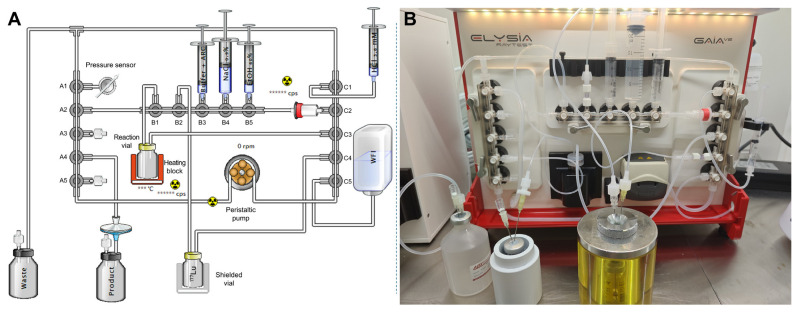
(**A**) Software system for the automated preparation of [^177^Lu]Lu-PSMA-ALB-56 with the GAIA^®^ synthesizer according to the optimized method; (**B**) Cassette setup on the GAIA^®^ module for the synthesis of [^177^Lu]Lu-PSMA-ALB-56 according to the optimized method.

Given the results obtained using the automated synthesis protocol described above, a second method for preparing [^177^Lu]Lu-PSMA-ALB-56 was developed, incorporating a terminal purification step via solid-phase extraction (SPE) (Figure 10). In addition, the amount of AR compound used in the reaction was significantly increased, and a second AR compound was introduced in the final formulation of the radiopharmaceutical. Specifically, PSMA-ALB-56 vector was solubilized in a mixture of sodium acetate 0.8 M (250 µL), methionine 30 mg/mL (1.5 mL) and HCl 0.1 M (0.65 mL). This solution was transferred to a 3 mL syringe and connected at B3 position. Then, only 1 mL of HCl 3.6 mM pH 4.5 was conditioned in a 3 mL syringe and connected at C1 as a rinsing solution for the ^177^Lu vial. For optimal solid phase extraction (SPE) cartridge elution, 3 mL of ethanol 60% were conditioned in a 5 mL syringe and connected at B5. For both elution and formulation, 17.2 mL of saline were conditioned in a 20 mL syringe and connected at B4. Of note, it was found important to leave approximately 1 mL of air above the liquid in the syringes, in order to ensure complete recovery of their content. Finally, SPE cartridge (Sep-Pak C_18_ Plus Short, Waters, Milford, MA, USA) was used to connect position B5 horizontal to position C2 after appropriate manual preconditioning of the cartridge with 5 mL of absolute ethanol and 5 mL of WFI. The WFI bottle at C5 and the vial containing ^177^Lu were prepared and connected in the same way as in the first method. Finally, prior to sequence start, 0.5 mL of a 50 mg/mL ascorbic acid and 100 mg/mL sodium ascorbate solution was conditioned in a 3 mL syringe and injected manually into the terminal vial through a 0.22 µ filter.

The initial steps of this automated sequence were identical to those of the previous method. Then, after 15 min of radiolabeling at 95 °C, the content of the reaction vial was cooled with a few milliliters of WFI from the bag at C5 and transferred to the SPE cartridge. The reaction vial was then rinsed with WFI, which was also transferred to the SPE cartridge. The cartridge was subsequently eluted into the terminal vial using 4 successive fractions of ethanol 60% (3 mL in total) and saline. Finally, the remainder of the saline syringe was passed through the SPE cartridge and used to formulate the radiopharmaceutical. Table 3 summarizes the successive steps of the two automated radiolabeling methods described and provides a comparison between the protocols.

### 3.5. Radio-HPLC Method for Radiochemical Purity Determination

Radio-HPLC analyses were conducted on a Nexera X3 system (Shimadzu, Kyoto, Japan) with HPLC-grade solvents. The radio-HPLC setup included a solvent degasser (DGU-405), a solvent pump (LC40D), an autosampler (SIL-40) with a 20 µL injection volume, a column oven (CTO-40S) set at 30 °C, a UV detector (SPD-40 190–700 nm) set at 254 and 280 nm, and a radioactivity detector (GABI Nova with mid-energy probe and 2 × 5 µL flow cell) connected in series. For radiation detection, a wide channel covering energies from 10 to 2000 keV was used. The column was a C_18_ ACE^®^ Equivalence™, 3.0 × 150 mm, 110 Å pore size and 3 μm particle size. The flow rate was maintained at 0.6 mL/min, and the mobile phase gradient was programmed as follows: from 0.1% TFA in water (A) to 0.1% TFA in acetonitrile (B): 0–2.5 min 95/5 A/B; 2.5–8.5 min linear gradient from 95/5 A/B to 20/80 A/B; 8.5–11 min 20/80 A/B; 11–12 min linear gradient from 20/80 A/B to 95/5 A/B; 12–14.5 min 95/5 A/B. The appropriate acquisition and analysis software (Gina Star 10, Elysia-Raytest, Straubenhardt, Germany) was used.

The identities of the impurities, ^177^Lu^3+^ and ^177^Lu colloids, were confirmed by independent injections of reference solutions (^177^Lu in acidic solution and ^177^Lu in alkaline solution, respectively), followed by comparison of their retention times with those observed in the reaction products.

### 3.6. Additional Quality Controls on Automated Synthesis Productions

The pH of the final [^177^Lu]Lu-PSMA-ALB-56 preparations, whether synthesized manually or automatically, was checked using 2-zones Rota pH 1–11 indicator paper (VWR, Radnor, PA, USA).

For ^177^Lu radionuclide identification, a gamma-spectrometry analysis was conducted on a low-activity sample of each [^177^Lu]Lu-PSMA-ALB-56 automated synthesis product using a Hidex AMG^®^ (LabLogic, Sheffield, UK) gamma counter. This test aimed to measure the emitted energies and compare with a ^177^Lu reference spectrum [97], with a special focus on the 208 keV and 113 keV peaks from gamma photons, as specified by the European Pharmacopoeia [98].

Half-life of each [^177^Lu]Lu-PSMA-ALB-56 automated synthesis product was determined by repeated measurements of low activity samples over 12 weeks (1 weekly measurements per batch), aiming for a half-life result within the expected range of 5.982 to 7.312 days and with an anticipated value of 6.647 days [99].

Although the ^177^Lu production and purification process is not expected to result in the presence of radioactive impurities, radionuclidic purity was determined on a sample of each [^177^Lu]Lu-PSMA-ALB-56 automated synthesis product. First, the possible traces of ^175^Yb (t_1/2_ = 4.18 days; can be formed from the neutron activation of ^174^Yb present as a minor impurity in the enriched ^176^Yb target) were investigated in the samples by performing a gamma spectrum and checking for the characteristic gamma peaks of this radioelement (i.e., 283, and 396 keV). Similarly, the trace level of ^177m^Lu potentially co-produced with ^177^Lu was determined by recording the gamma ray spectra of the sample aliquot, initially having high radioactive concentration, after complete decay of ^177^Lu activity (8–10 t_1/2_ of ^177^Lu, i.e., for a period of 50–70 days). The characteristics gamma peaks of ^177m^Lu are 128, 153, 228, 378, 414 and 418 keV.

## 4. Conclusions

From a general perspective, the screening of radiolabeling conditions with ^177^Lu can be downscaled and performed at low activities to identify the most suitable reagents (for example, reaction buffers) for a given vector molecule. However, conditions optimized in this way must be systematically validated on scaled-up reactions involving high activities, particularly when an automated process is intended. This work highlights the critical role of antioxidant compounds in the preparation and formulation of ^177^Lu-based radiopharmaceuticals, with particular emphasis on the need for high concentrations to ensure effective protection against radiolysis. Ultimately, the optimal radiolabeling conditions for the ABM-bearing vector PSMA-ALB-56 were successfully identified and adapted for use in an original automated synthesis protocol, potentially facilitating the clinical translation of this TRT vector with improved pharmacokinetic properties. Importantly, the automated protocol presented here also has the potential to be adapted for the radiolabeling of other ^177^Lu-based radiopharmaceutical candidates.

## Figures and Tables

**Table 1:** Quality control results for three representative batches of [^177^Lu]Lu-PSMA-ALB-56 produced under optimized automated conditions.

Test	Batch 1	Batch 2	Batch 3
**Appearance**	Clear, colorless solution	Clear, colorless solution	Clear, colorless solution
**Identification**			
Energy of gamma photons (keV)	113 and 208	113 and 208	113 and 208
Half-life (days)	6.86 ± 0.16	6.76 ± 0.10	7.00 ± 0.31
**pH**	4.5	4.5	4.5
**Radionuclidic purity**			
(^177^Lu) Lutetium (%)	100	100	100
γ-Emitting impurities	None identified	None identified	None identified
**Radiochemical purity (HPLC)**			
[^177^Lu]Lu-PSMA-ALB-56 (%)	98.96	99.02	98.79
[^177^Lu]lutetium impurities (%)	1.04	0.98	1.21
**Filter integrity test**	>3500 mbar	>3500 mbar	>3500 mbar
**Volume activity at EoS (MBq/mL)**	101.49	100.99	91.78
**Specific activity at EoS (MBq/µg)**	31.21	31.08	28.18
**Molar activity at EoS (GBq/µmol)**	41.53	41.35	37.49
**Ethanol amount (%, calculated)**	~8.9	~8.9	~8.9
**Ascorbic acid concentration (mg/mL, calculated)**	~3.6	~3.6	~3.6
**Radiochemical yield (%)** (Based on RCP determined by HPLC)	79.46	81.85	78.05
**Stability over 120 h (HPLC)**	≥95%	≥96%	≥96%

**Table 2:** Buffer and AR compound solutions used in the manual [^177^Lu]Lu-PSMA-ALB-56 radiolabeling assays.

Buffer	Molarity	pH	Volume Set for Reaction	References
HEPES	1.25 M	4.5	160 µL	[91]
Ascorbate	1.8 M	4.5	160 µL	[75]
Ammonium acetate	0.1 M	4.5	160 µL	[92,93]
Ammonium acetate	1 M	4.5	160 µL	[94]
Sodium acetate	0.1 M	4.5	160 µL	[95]
Sodium acetate	0.5 M	4.5	160 µL	[11]
**AR compound**	**Concentration (initial)**		**Volume added to reaction**	**References**
Ascorbic acid	13 mg/mL		10 µL	[54,65]
Gentisic acid	12 mg/mL		10 µL	[96]
Cysteine	36 mg/mL		10 µL	[53]
Methionine	30 mg/mL		10 µL	[64]

**Table 3:** General [^177^Lu]Lu-PSMA-ALB-56 automated synthesis steps.

	*Initial Protocol Without SPE Purification*
1	Kit integrity test (pressurization > 1500 mbar and pressure reduction of no more than 400 mbar over 20 s).
2	Transfer of PSMA-ALB-56 solubilized in buffer to the ^177^Lu vial, then transfer back to the reaction vial.
3	Pre-heating of the reaction vial (60 °C).
4	Rinsing of the ^177^Lu vial with 1.4 mL HCl 3.6 mM, then transfer back to the reaction vial.
5	Tubing purge with filtered air.
6	Labeling at 95 °C during 15 min.
7	During labeling, rinsing and purge of manifold B.
8	End of radiolabeling: addition of 3 mL DTPA 1 mg/mL to the reaction medium.
9	Transfer of the reaction mixture from the reaction vial to the product vial
10	Reaction vial washing with 7 mL DTPA 1 mg/mL and transfer from the reaction vial to the product vial.
11	Product vial release and filter integrity testing
12	Closing all valves, end of synthesis
	***Optimized protocol with SPE purification***
1	Kit integrity test (pressurization > 1500 mbar and pressure reduction of no more than 400 mbar over 20 s).
2	Transfer of PSMA-ALB-56 solubilized in buffer to the ^177^Lu vial, then transfer back to the reaction vial.
3	Pre-heating of the reaction vial (60 °C).
4	Rinsing of the ^177^Lu vial with 1.0 mL WFI, then transfer back to the reaction vial.
5	Tubing purge with filtered air.
6	Labeling at 95 °C during 15 min.
7	Transfer of the reaction mixture from the reaction vial to the SPE cartridge
8	Washing of the reaction vial with 10 mL WFI and transfer from the reaction vial onto the SPE cartridge
9	SPE cartridge rinsing with WFI and purging of the tubing with filtered air
10	[^177^Lu]Lu-PSMA-ALB-56 elution to the terminal vial with alternating ethanol 60% (total 3 mL) and saline
11	Formulation of the final product with remaining NaCl 0.9% (total elution + formulation = 17.2 mL)
12	Product vial release and filter integrity testing
13	Closing all valves, end of synthesis

## Data Availability

The original contributions presented in this study are included in the article and Supplementary Materials. Further inquiries can be directed to the corresponding author.

## Supplementary Materials

The following supporting information can be downloaded at: https://www.mdpi.com/article/10.3390/ijms26199642/s1.
