# Supplementary material for: Tailored Reaction Conditions and Automated Radiolabeling of [177Lu]Lu-PSMA-ALB-56 in a 68Ga Setting: The Critical Impact of Antioxidant Concentrations
Source: Int J Mol Sci. 2025 Oct 2;26(19):9642. doi: 10.3390/ijms26199642 (PMC12525066; doi:10.3390/ijms26199642)

Tailored Reaction Conditions and Automated Radiolabeling of [^{177}Lu]Lu-PSMA-ALB-56 in a ^{68}Ga Setting: The Critical Impact of Antioxidant Concentrations

Johanne Vanney [1], Léa Rubira [1], Jade Torchio [1] and Cyril Fersing [1,2,*]

[1] Nuclear medicine department, Institut régional du Cancer de Montpellier (ICM), Univ. Montpellier, Montpellier, France
[2] IBMM, Univ Montpellier, CNRS, ENSCM, Montpellier, France
* Correspondence: cyril.fersing@umontpellier.fr; Tel.: +33 4 67 61 24 78

Supplementary Materials Data

Table of content

SI – Detailed automated synthesis sequences for [^{177}Lu]Lu-PSMA-ALB-56 radiolabeling 1
SII – Quality controls of the commercial PSMA-ALB-56 vector molecule 4
SIII – Radiochemical purities in HPLC of manual radiolabeling assays 8
SIV – Radiochemical purity determined by HPLC for [^{177}Lu]Lu-PSMA-ALB-56 radiolabeling 9
SV – Gamma-spectrometry analyses of the [^{177}Lu]Lu-PSMA-ALB-56 test batches 11
SVI – Half-life determination conducted on the [^{177}Lu]Lu-PSMA-ALB-56 test batches 12
SVII – Radionuclide purity estimation of the [^{177}Lu]Lu-PSMA-ALB-56 test batches 14

SI – Detailed automated synthesis sequences for [^{177}Lu]Lu-PSMA-ALB-56 radiolabeling

A. Initial protocol without SPE purification

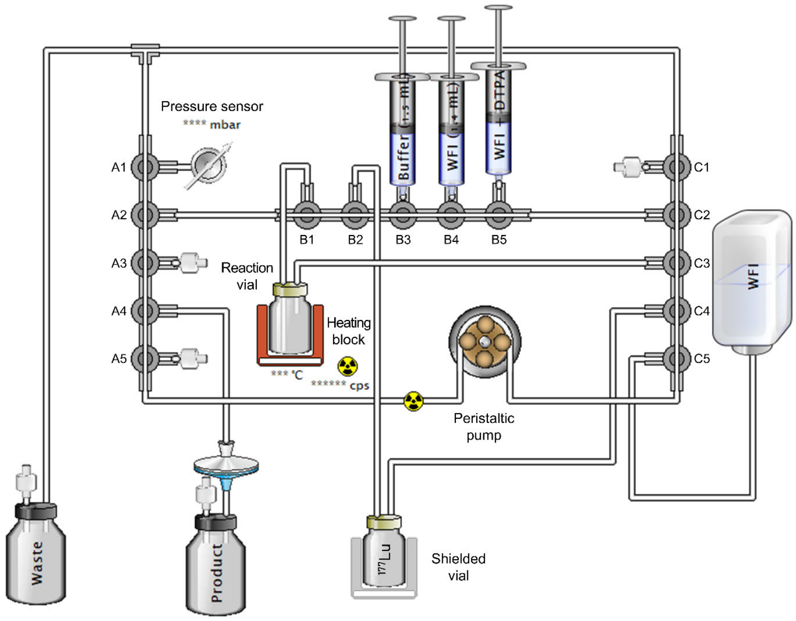

	A1	A2	A3	A4	A5	B1	B2	B3	B4	B5	C1	C2	C3	C4	C5	Pump	Heater	Lift	Heater	Wait Condition	Wait Timeout	Measurement	Description (English)
**0**	0	0	0	0	0	0	0	0	0	0	0	0	0	0	0	off	off	0.0	off				Initial State
**1**	1	2	0	0	1	2	0	0	0	0	0	3	2	0	0	250	off	0.0	off	Pressure > 1500mbar	180		Kit Integrity Test: Pressurizing
**2**	1	2	0	0	0	2	0	0	0	0	1	2	2	0	0	off	off	0.0	off	Fixed delay	5		Kit Integrity Test: Equilibration
**3**	1	2	0	0	0	2	0	0	0	0	1	2	2	0	0	off	off	0.0	off	Fixed delay	15	Gaia–Pressure changes less than 400.0mbar	Kit Integrity Test: Measuring
**4**	2	2	2	0	0	0	0	0	0	0	2	2	0	0	0	off	off	0.0	off	Fixed delay	5		Kit Integrity Test: Venting
**5**	0	0	1	0	0	0	1	0	3	0	2	2	0	3	0	-200	off	0.0	off	Fixed delay	25		Lu dilution 1
**6**	0	0	1	0	0	0	1	0	0	0	3	1	0	3	0	-200	off	0.0	off	Fixed delay	5		Lu dilution 2
**7**	0	0	1	0	0	1	3	0	0	0	3	0	1	3	0	250	off	0.0	off	Fixed delay	30		Lu transfert
**8**	0	0	1	0	0	1	1	3	0	0	0	0	0	3	0	-200	off	0.0	off	Fixed delay	35		Buffer addition 1
**9**	0	0	1	0	0	1	3	0	0	0	3	0	1	3	0	250	60	0.0	off	Fixed delay	30		Buffer transfert
**10**	0	0	1	0	0	1	3	0	0	0	3	0	3	0	0	-200	60	0.0	off	Fixed delay	8		B1 purge
**11**	0	0	1	0	0	1	3	0	0	0	3	0	2	0	0	off	60	0.0	off	Fixed delay	3		Venting
**12**	0	0	0	0	0	0	3	0	0	0	0	0	0	0	0	off	98	0.0	off	Fixed delay	1	Gaia–Reactor Detector	Activity measurement
**13**	0	0	0	0	0	0	3	0	0	0	0	0	0	0	0	off	98	0.0	off	Fixed delay	5		Labeling part 1a
**14**	0	0	1	0	0	1	0	0	0	0	3	1	3	0	0	-100	98	0.0	off	Fixed delay	10		B1 line purge
**15**	1	1	0	0	0	0	0	0	0	0	0	1	0	0	3	-150	98	0.0	off	Fixed delay	25		Rinsing
**16**	0	0	0	0	0	0	0	0	0	0	3	0	0	0	0	-150	98	0.0	off	Fixed delay	15		Purge 1
**17**	0	3	0	0	0	0	0	0	0	0	0	3	0	0	0	150	98	0.0	off	Fixed delay	5		Purge 2a
**18**	0	3	1	0	0	0	0	0	0	0	0	3	0	0	0	150	98	0.0	off	Fixed delay	20		Purge 2b
**19**	0	0	0	0	0	0	0	0	0	0	0	0	0	0	0	off	98	0.0	off	Fixed delay	820		Labelling part 1b
**20**	0	0	1	0	0	1	0	0	0	3	0	0	3	0	0	-200	98	0.0	off	Fixed delay	12		3mL DTPA addition
**21**	0	0	1	0	0	1	0	0	0	0	3	1	3	0	0	-75	98	0.0	off	Fixed delay	15		DTPA purge
**22**	0	0	0	0	0	0	3	0	0	0	0	0	0	0	0	off	98	0.0	off	Fixed delay	300		Labelling part 2
**23**	0	1	0	3	1	3	0	0	0	0	0	1	3	0	0	250	off	0.0	off	Fixed delay	35		Transfert to terminal vial 1
**24**	0	1	0	3	1	1	0	0	0	3	0	0	3	0	0	-200	off	0.0	off	Fixed delay	30		Rinsing Reactor 1
**25**	0	1	0	3	1	3	0	0	0	0	0	1	3	0	0	250	off	0.0	off	Fixed delay	60		Transfert to terminal vial 2
**26**	0	1	0	3	1	3	0	0	0	0	0	1	3	0	0	off	off	0.0	off	Fixed delay	1	Gaia–Reactor Detector	Measuring Empty Reactor Activity
**27**	1	1	2	2	2	0	0	0	0	0	0	2	0	0	0	off	off	0.0	off	Fixed delay	2		Venting system
**28**	1	2	0	0	0	0	0	0	0	0	0	2	0	0	0	off	off	0.0	off	User prompt	1		Withdraw terminal vial, connect ter
**29**	0	0	0	1	0	0	0	0	0	0	0	0	0	0	3	-250	off	0.0	off	Fixed delay	30		Filter integrity test: filter purge
**30**	1	0	0	0	0	0	0	0	0	0	0	0	0	0	0	250	off	0.0	off	Fixed delay	5		Filter integrity test: line purge prep
**31**	1	0	1	0	0	0	0	0	0	0	0	0	0	0	0	250	off	0.0	off	Fixed delay	15		Filter integrity test: line purge 1
**32**	0	3	1	0	0	0	0	0	0	0	0	3	0	0	0	250	off	0.0	off	Fixed delay	20		Filter integrity test: line purge 2
**33**	0	3	0	0	0	0	0	0	0	0	0	3	0	0	0	-250	off	0.0	off	Fixed delay	10		Filter integrity test: line purge 3
**34**	1	0	0	2	0	0	0	0	0	0	0	0	0	0	0	-250	off	0.0	off	Pressure > 2500mbar	120		Filter integrity test: pressurizing
**35**	1	0	0	2	0	0	0	0	0	0	0	0	0	0	0	-150	off	0.0	off	Pressure > 2900mbar	200		Filter integrity test: approaching bu
**36**	1	0	0	2	0	0	0	0	0	0	0	0	0	0	0	-120	off	0.0	off	Sterile Filter Test	600		Filter integrity test: measuring bubb
**37**	2	0	2	2	0	0	0	0	0	0	0	2	2	0	0	off	off	0.0	off	Fixed delay	3		Venting
**38**	0	0	0	0	0	0	0	0	0	0	0	0	0	0	0	off	off	0.0	off	Stop Synthesis	5		Stop

B. Optimized protocol with SPE purification

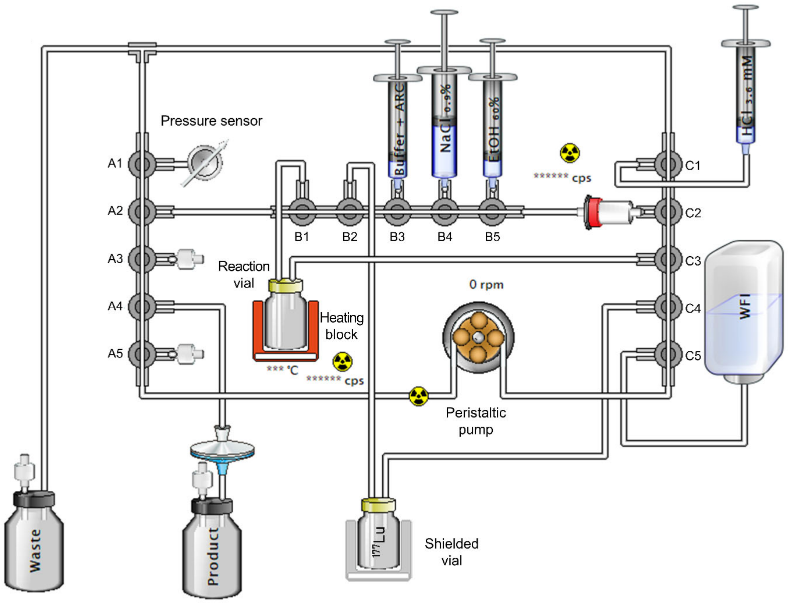

	A1	A2	A3	A4	A5	B1	B2	B3	B4	B5	C1	C2	C3	C4	C5	Pump	Heater	Lift	Heater	Wait Condition	Wait Timeout	Measurement	Description (English)
0	0	0	0	0	0	0	0	0	0	0	0	0	0	0	0	off	off	0.0	off				Initial State
1	0	3	0	0	1	0	0	0	0	0	0	3	0	0	0	250	off	0.0	off	Fixed delay	10		C18 initial purge
2	1	2	0	0	1	2	0	0	0	0	0	3	2	0	0	250	off	0.0	off	Pressure > 1500mbar	180		Kit Integrity Test: Pressurizing
3	1	2	0	0	0	2	0	0	0	0	0	3	2	0	0	off	off	0.0	off	Fixed delay	5		Kit Integrity Test: Equilibration
4	1	2	0	0	0	2	0	0	0	0	0	3	2	0	0	off	off	0.0	off	Fixed delay	15	Gaia–Pressure changes less than 400.0mbar	Kit Integrity Test: Measuring
5	2	2	2	0	0	0	0	0	0	0	0	2	0	0	0	off	off	0.0	off	Fixed delay	4		Kit Integrity Test: Venting
6	0	0	0	0	1	0	1	3	0	0	2	2	0	3	0	-250	off	0.0	off	Fixed delay	40		Buffer addition in Lu
7	0	0	3	0	1	1	3	0	0	0	0	0	1	3	0	250	off	0.0	off	Fixed delay	40		Lu in buffer transfert
8	0	0	0	0	0	1	1	3	0	0	3	0	0	0	0	-70	60	0.0	off	Fixed delay	15		Lu vial rinsing 1
9	0	1	0	0	0	0	3	0	0	0	0	0	0	3	0	-75	60	0.0	off	Fixed delay	20		Lu vial rinsing 2
10	1	0	0	0	1	1	3	0	0	0	0	0	1	3	0	250	80	0.0	off	Fixed delay	30		Rinsing WFI transfert
11	0	0	3	0	1	1	3	0	0	0	0	0	3	0	0	off	97	0.0	off	Fixed delay	2		B1 purge preparation
12	0	0	3	0	1	1	3	0	0	0	0	0	3	0	0	-200	97	0.0	off	Fixed delay	20		B1 purge
13	0	0	3	0	1	0	3	0	0	0	0	0	2	0	0	off	97	0.0	off	Fixed delay	3		Venting
14	0	0	0	0	0	0	0	0	0	0	0	0	0	0	0	off	97	0.0	off	Fixed delay	1	Gaia–Reactor Detector	Activity measurement
15	0	0	0	0	0	0	0	0	0	0	0	0	0	0	0	off	96	0.0	off	Fixed delay	375		Labeling part 1
16	1	1	0	0	0	0	0	0	0	0	0	1	0	0	3	-150	96	0.0	off	Fixed delay	30		Rinsing and cartridge activation
17	1	3	0	0	0	0	0	0	0	0	0	0	0	0	0	200	96	0.0	off	Fixed delay	5		Purge 1
18	1	3	1	0	0	0	0	0	0	0	0	0	0	0	0	200	96	0.0	off	Fixed delay	10		Purge 2
19	0	3	1	0	0	0	0	0	0	0	0	3	0	0	0	200	96	0.0	off	Fixed delay	10		Purge 3
20	0	1	3	0	1	0	0	0	0	0	0	0	3	0	0	-200	96	0.0	off	Fixed delay	10		B1 line purge 1
21	0	0	0	0	0	0	0	0	0	0	0	0	0	0	0	off	96	0.0	off	Fixed delay	375		Labelling part 2
22	1	1	0	0	0	3	0	0	0	0	0	0	1	0	3	-160	38	0.0	off	Fixed delay	12		Delution
23	1	1	0	0	0	1	0	0	0	0	0	1	3	0	0	250	38	0.0	off	Fixed delay	10		C18 trapping: preparation
24	1	1	1	0	0	1	0	0	0	0	0	1	3	0	0	100	38	0.0	off	Fixed delay	60		C18 trapping
25	1	1	0	0	0	3	0	0	0	0	0	0	1	0	3	-250	38	0.0	off	Fixed delay	15		Rinsing reactor
26	1	1	0	0	0	1	0	0	0	0	0	1	3	0	0	250	38	0.0	off	Fixed delay	10		C18 rinsing: preparartion
27	1	1	1	0	0	1	0	0	0	0	0	1	3	0	0	200	38	0.0	off	Fixed delay	60		C18 rinsing
28	0	3	1	0	0	0	0	0	0	0	0	3	0	0	0	250	38	0.0	off	Fixed delay	5	Gaia–Reactor Detector	C18 purge/Measuring empty reactor activity
29	0	3	1	0	0	0	0	0	0	0	0	3	0	0	0	250	38	0.0	off	Fixed delay	5	Gaia–Manifold Detector 2	C18 purge/Measuring cartrige activity
30	0	3	1	1	0	0	0	0	0	1	0	3	0	0	0	-15	off	0.0	off	Fixed delay	35		C18 elution: EtOH 1
31	0	3	1	1	0	0	0	0	1	0	0	3	0	0	0	-15	off	0.0	off	Fixed delay	20		C18 elution: NaCl 1
32	0	3	1	1	0	0	0	0	1	1	0	3	0	0	0	-15	off	0.0	off	Fixed delay	30		C18 elution: EtOH 2
33	0	3	1	1	0	0	0	0	1	0	0	3	0	0	0	-15	off	0.0	off	Fixed delay	10		C18 elution: NaCl 2
34	0	3	1	1	0	0	0	0	1	1	0	3	0	0	0	-15	off	0.0	off	Fixed delay	30		C18 elution: EtOH 3
35	0	3	1	1	0	0	0	0	1	0	0	3	0	0	0	-15	off	0.0	off	Fixed delay	10		C18 elution: NaCl 3
36	0	3	1	1	0	0	0	0	1	1	0	3	0	0	0	-15	off	0.0	off	Fixed delay	40		C18 elution: EtOH 4
37	0	3	1	1	0	0	0	0	1	0	0	3	0	0	0	-150	off	0.0	off	Fixed delay	110		Formulation
38	0	1	3	1	0	0	0	0	0	0	0	3	0	0	0	-200	off	0.0	off	Fixed delay	20	Gaia–Manifold Detector 2	Formulation/Measuring C18 activity post-elution
39	1	1	3	1	0	0	0	0	0	0	0	3	0	0	0	off	off	0.0	off	Fixed delay	3		Venting preparation
40	1	2	0	0	0	0	0	0	0	0	0	2	0	0	0	off	off	0.0	off	User prompt	1		Withdraw terminal vial, connect termina filter on waste vial and retire venting filter
41	0	0	0	1	0	0	0	0	0	0	0	0	0	0	3	-250	off	0.0	off	Fixed delay	30		Filter integrity test: filter purge
42	1	0	0	0	0	0	0	0	0	0	0	0	0	0	0	250	off	0.0	off	Fixed delay	5		Filter integrity test: line purge preparation
43	1	0	1	0	0	0	0	0	0	0	0	0	0	0	0	250	off	0.0	off	Fixed delay	15		Filter integrity test: line purge 1
44	0	3	1	0	0	0	0	0	0	0	0	3	0	0	0	250	off	0.0	off	Fixed delay	20		Filter integrity test: line purge 2
45	0	3	0	0	0	0	0	0	0	0	0	3	0	0	0	-250	off	0.0	off	Fixed delay	10		Filter integrity test: line purge 3
46	1	0	0	2	0	0	0	0	0	0	0	0	0	0	0	-250	off	0.0	off	Pressure > 2500mbar	120		Filter integrity test: pressurizing
47	1	0	0	2	0	0	0	0	0	0	0	0	0	0	0	-150	off	0.0	off	Pressure > 2900mbar	200		Filter integrity test: approaching bubble point value
48	1	0	0	2	0	0	0	0	0	0	0	0	0	0	0	-120	off	0.0	off	Sterile Filter Test	600		Filter integrity test: measuring bubble point value
49	2	0	2	2	0	0	0	0	0	0	0	2	2	0	0	off	off	0.0	off	Fixed delay	4		Venting
50	0	0	0	0	0	0	0	0	0	0	0	0	0	0	0	off	off	0.0	off	Stop Synthesis	5		Stop

SII – Quality controls of the commercial PSMA-ALB-56 vector molecule

CAS NO.: 2306049-48-7

S A M P L E   I N F O R M A T I O N

Sample Name:	ZYH21798-003-10_1	Acquired By:	QFF2306
Sample Type:	Unknown	Sample Set Name:	HY_770_1_20230327_3_138
Vial:	1:E,2	Acq. Method Set:	HY_770_H01RS_RP_HPLC_C2_25MIN
Injection #:	1	Processing Method:	HY_770_1
Injection Volume:	5.00 ul	Channel Name:	2998 Ch1 225nm@4.8nm
Run Time:	25.0 Minutes	Remark	
Date Acquired:	3/27/2023 2:52:38 PM CST	Column Number:	1MG/ML ACN:H2O=50:50 A-RP-1141
Date Processed:	3/27/2023 3:20:47 PM CST	Processed By:	QFF2306/group_leader

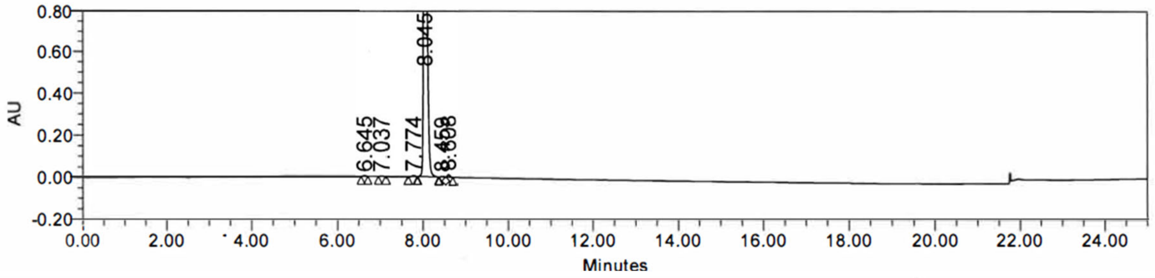

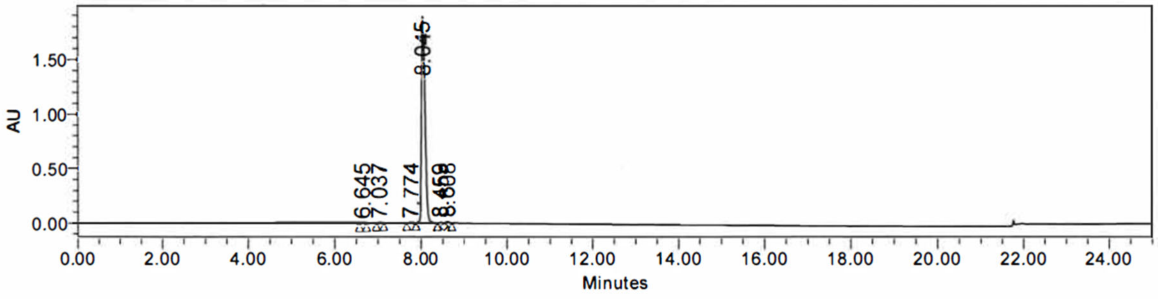

	RT	Height	Width (sec)	Area	% Area
1	6.645	2882	9.600	11041.3781	0.1022
2	7.037	2394	10.000	10258.9655	0.0949
3	7.774	5292	12.400	22495.5283	0.2081
4	8.045	1889439	29.400	10691004.8379	98.9153
5	8.459	3507	9.200	18310.0104	0.1694
6	8.608	12569	10.400	55129.5677	0.5101
Sum				10808240.3	100.0000

CAS NO.: 2306049-48-7

SAMPLE INFORMATION

Sample Name:	ZYH21798-003-10_2	Acquired By:	QFF2306
Sample Type:	Unknown	Sample Set Name:	HY_770_1_20230327_3_138
Vial:	1:E,3	Acq. Method Set:	HY_770_H01RS_RP_HPLC_C2_25MIN
Injection #:	1	Processing Method:	HY_770_1
Injection Volume:	5.00 ul	Channel Name:	2998 Ch1 225nm@4.8nm
Run Time:	25.0 Minutes	Remark:	
Date Acquired:	3/27/2023 3:18:28 PM CST	Column Number:	1MG/ML ACN:H2O=50:50 A-RP-1141
Date Processed:	3/27/2023 3:47:34 PM CST	Processed By:	QFF2306/group_leader

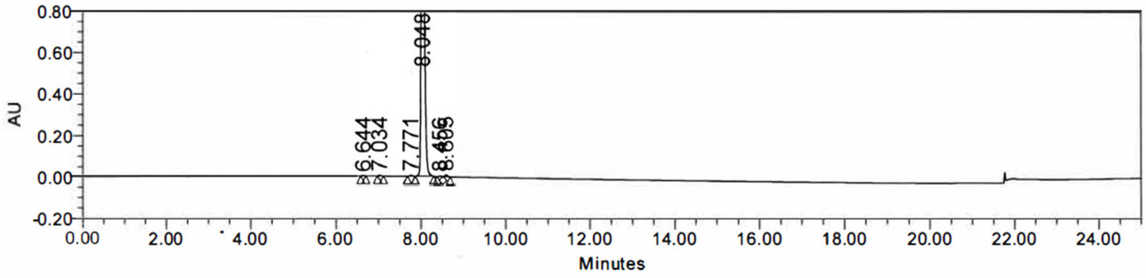

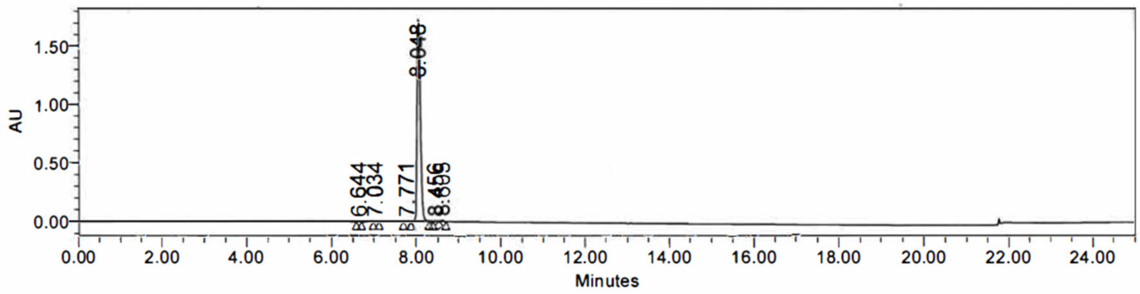

	RT	Height	Width (sec)	Area	% Area
1	6.644	2114	7.600	7679.9100	0.0829
2	7.034	3481	8.200	13165.6189	0.1421
3	7.771	4869	10.600	20890.6414	0.2255
4	8.048	1726672	25.800	9165603.8606	98.9203
5	8.456	2861	8.200	14615.7696	0.1577
6	8.605	9893	10.400	43690.3116	0.4715
Sum				9265646.1	100.0000

```
File ..2023-03-27\ZYH21798-003-10-LCMS-02-019306.D Tgt Mass (EZX):
Injection Date    : 27-Mar-23, 13:03:25              Seq. Line : 0
Sample Name       : ZYH21798-003-10              Location  : P2-B-02
Acq. Operator     : admin                                  Inj : 1
Spec. Reported    : MS Integration                   Inj Volume : 3 ul
Acq. Method       : F:\Chem32\1\methods\1-POS-3MIN.M
Analysis Method   : F:\Chem32\1\methods\1-POS-3MIN.M
CAS NO.      : 2306049-48-7  Project: HY-770-001KF
Method Info : Mobile Phase: A: water(0.01%TFA) B:ACN(0.01%TFA)
              Gradient: 5% to 95%B within 1.3 min
              Flow Rate :1.8ml/min
              Column :Waters Xbridge C18, 4.6*50mm,3.5um A-RP-1261
              Oven Temperature : 45C
```

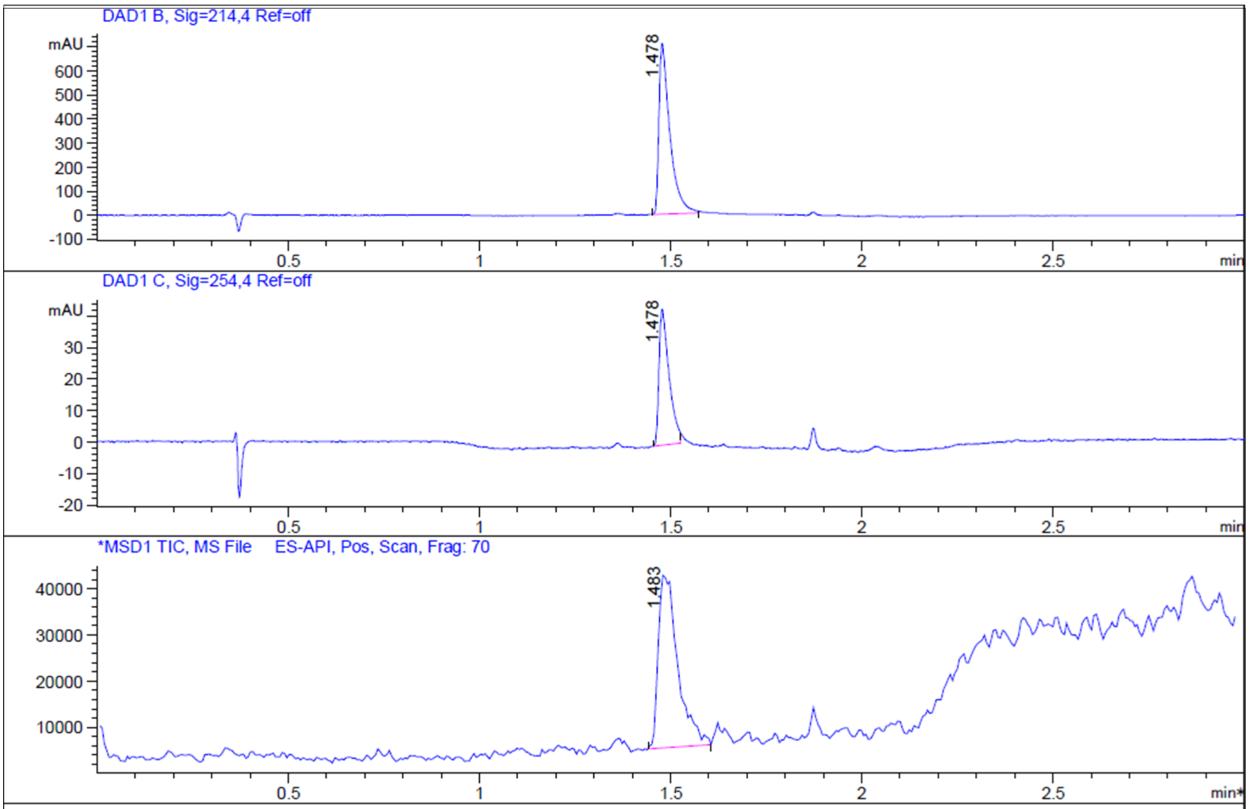

Integration Results for DAD1 B, Sig=214,4 Ref=off

RetTim	Width	Area	Height	Area%	MS(+)
1.48	0.03	1411.19	708.57	100.00	159

Integration Results for DAD1 C, Sig=254,4 Ref=off

RetTim	Width	Area	Height	Area%	MS(+)
1.48	0.03	79.32	43.26	100.00	159

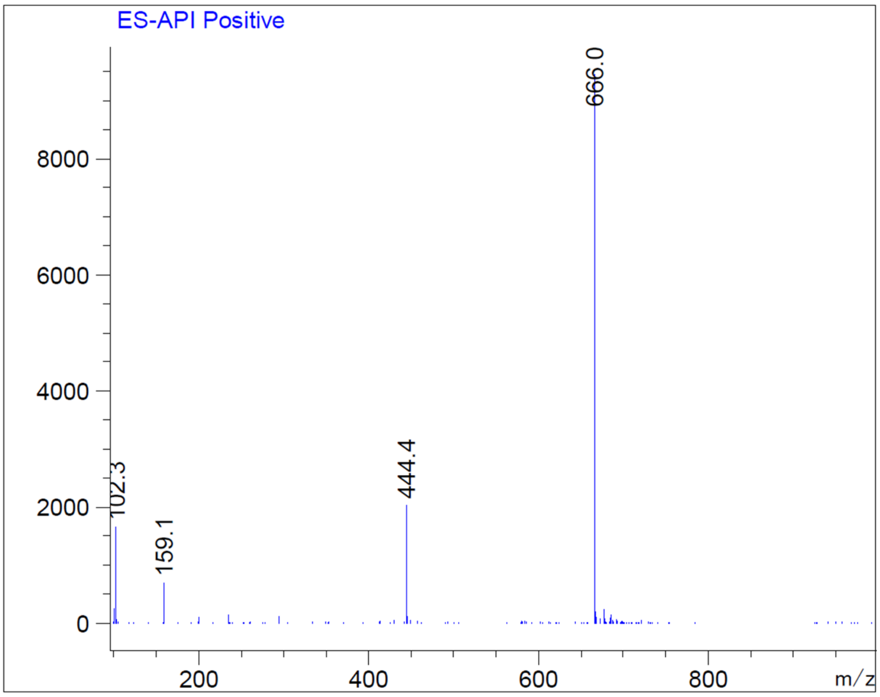
ES-API Positive
666.0
444.4
102.3
159.1
8000
6000
4000
2000
0
200
400
600
800
m/z

SIII – Radiochemical purities in HPLC of manual radiolabeling assays

	Assay 1	Assay 2	Assay 3	Mean (%)	%SD
Buffer solutions					
Ammonium acetate 0.1 M	99.50	95.31	89.56	94.79	4.99
Ammonium Acetate 1 M	99.80	97.10	94.59	97.16	2.61
Sodium ascorbate 1.8 M	98.09	95.23	89.61	94.31	4.31
HEPES 1.25 M	91.55	94.15	96.69	94.13	2.57
Sodium acetate 0.1 M	97.20	98.08	98.92	98.07	0.86
Sodium acetate 0.5 M	98.70	98.53	98.07	98.43	0.33
Antioxidant compound					
Ascorbic acid	95.76	97.26	94.88	95.97	1.20
Gentisic acid	95.14	99.78	97.03	97.32	2.33
Methionine	96.47	98.33	96.95	97.25	0.97
Cysteine	< 5	< 5	< 5	< 5	NC*

*NC = not calculated

SIV - Radiochemical purity determined by HPLC for [^{177}Lu]Lu-PSMA-ALB-56 radiolabeling

Test batch 1

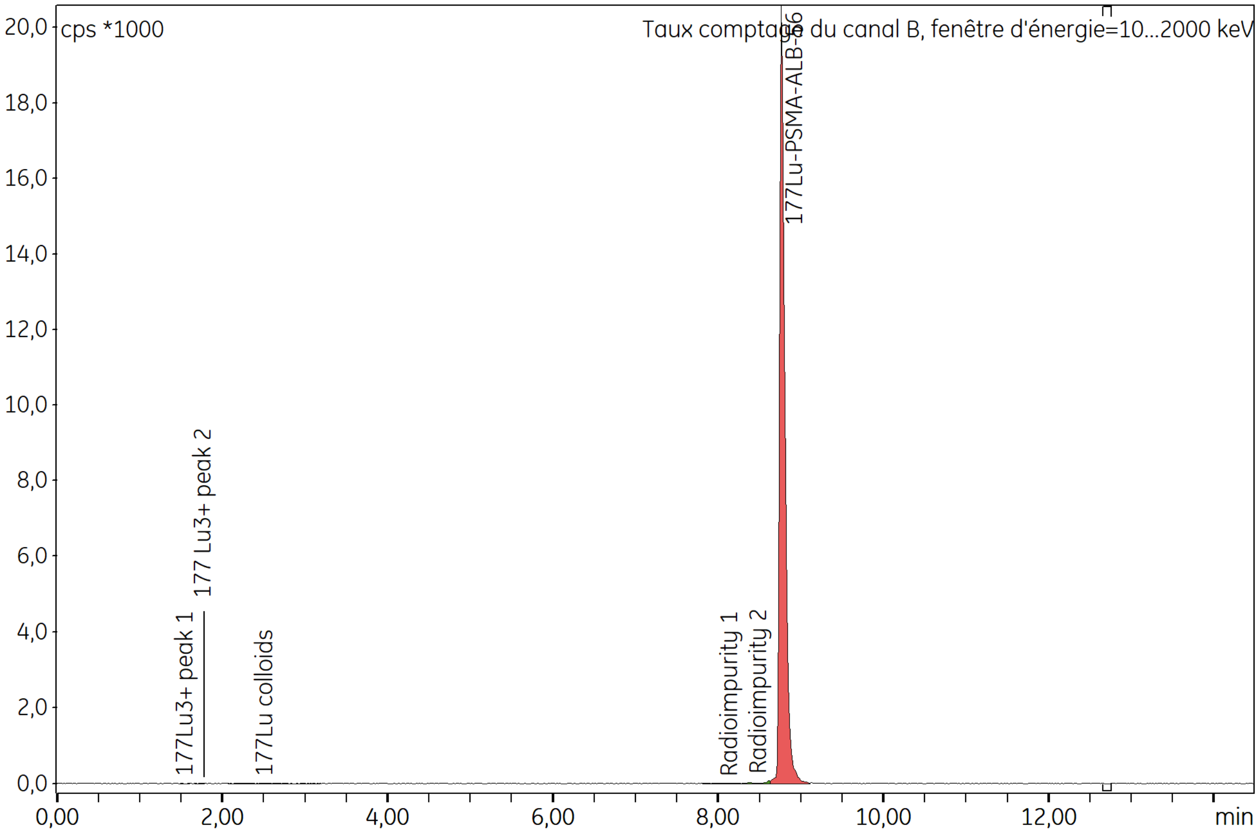

Test batch 2

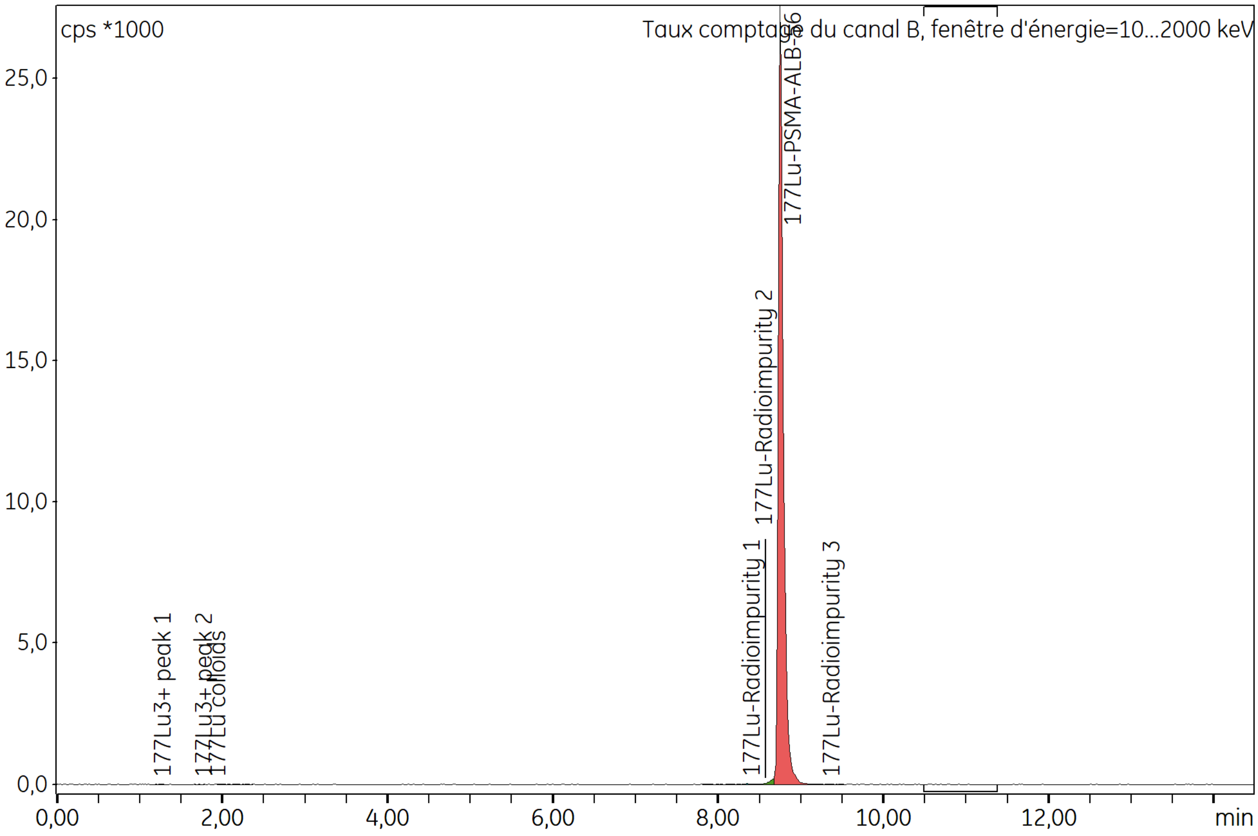

Test batch 3

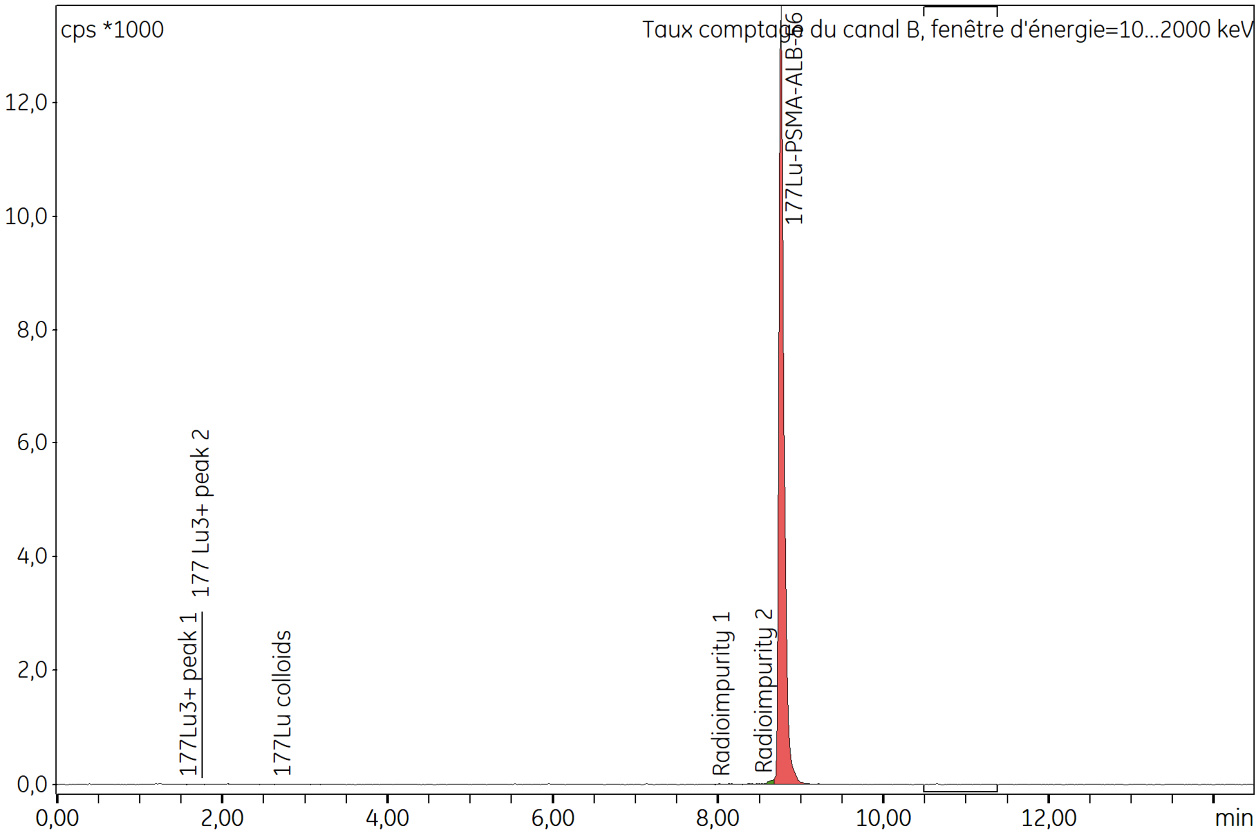

cps *1000
Taux comptage du canal B, fenêtre d'énergie=10...2000 keV
12,0
10,0
8,0
6,0
4,0
2,0
0,0
0,00
2,00
4,00
6,00
8,00
10,00
12,00
min
177Lu3+ peak 1
177 Lu3+ peak 2
177Lu colloids
Radioimpurity 1
Radioimpurity 2
177Lu-PSMA-ALB-56

SV – Gamma-spectrometry analyses of the [^{177}Lu]Lu-PSMA-ALB-56 test batches

Test batch 1

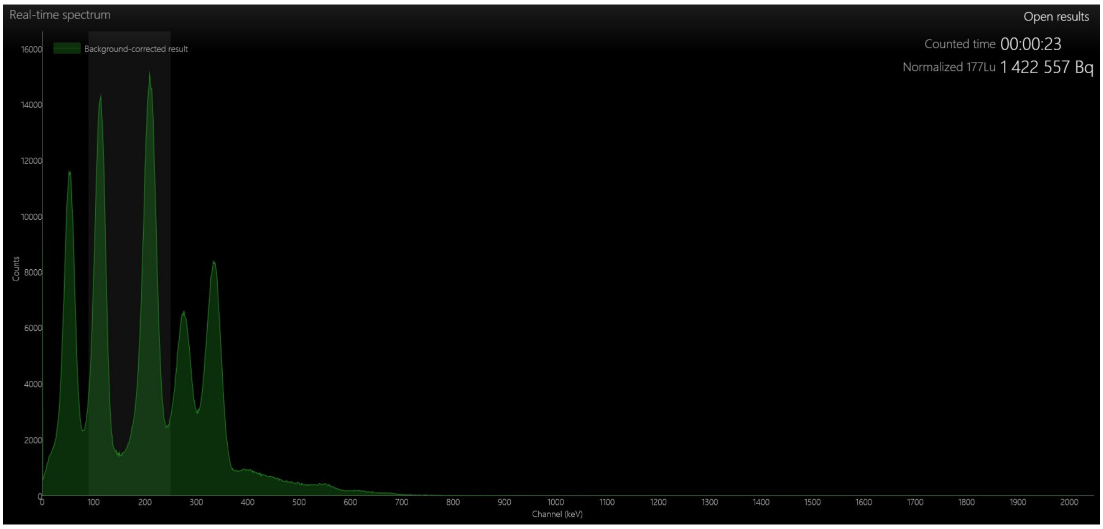

Test batch 2

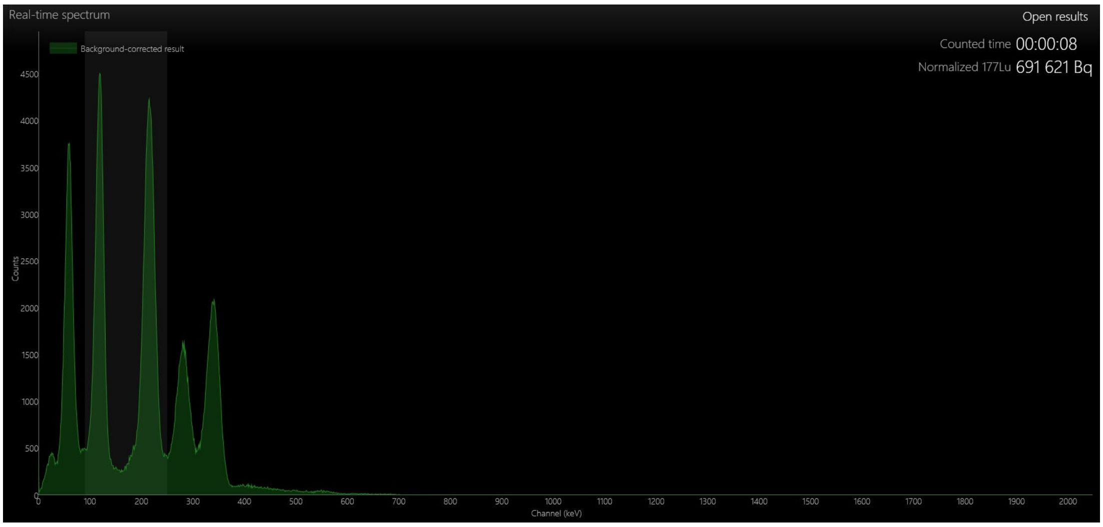

Test batch 3

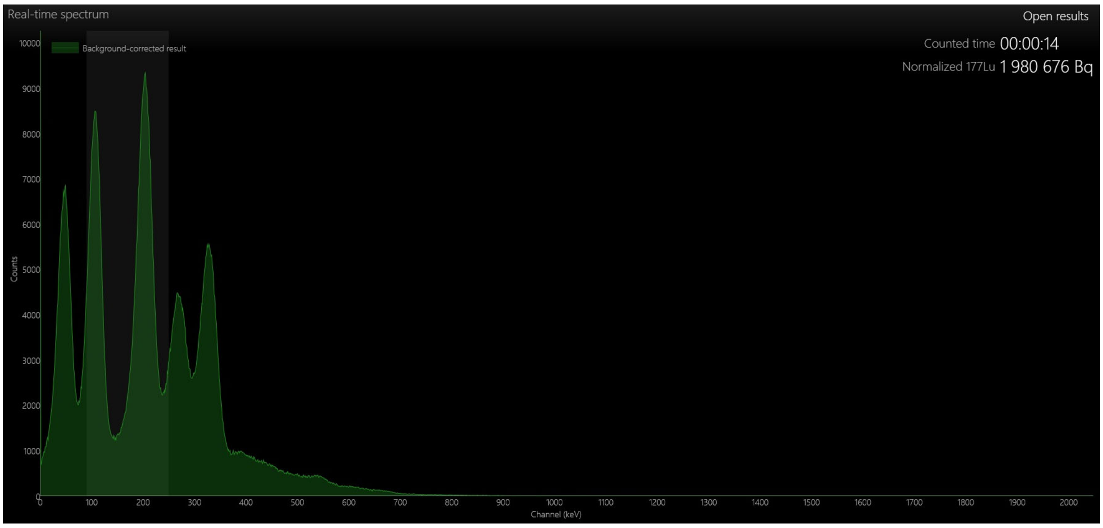

SVI – Half-life determination conducted on the [^{177}Lu]Lu-PSMA-ALB-56 test batches

Test batch 1

Time (min)	CPM	Calculated decay constant (day^{-1})	Calculated half-life (day)
16/07/2024 17:00	5 967 418	-	-
22/07/2024 14:06	3 402 575	0.0955504275	7.254255144
29/07/2024 10:13	1 710 435	0.0982548271	7.054586537
05/08/2024 09:21	840 824	0.0995725149	6.961230026
12/08/2024 10:13	405 625	0.1006315829	6.887968572
19/08/2024 15:18	192 197	0.1012573100	6.845403858
26/08/2024 08:50	95 081	0.1018045160	6.808609362
02/09/2024 09:46	45 580	0.1021952686	6.782576046
09/09/2024 18:54	20 946	0.1026183698	6.754611105
16/09/2024 15:31	10 159	0.1029368697	6.733711472
24/09/2024 17:26	4 528	0.1025986791	6.755907452
01/10/2024 17:48	2 067	0.1034355285	6.701248505
07/10/2024 13:59	1 197	0.1027373652	6.746787593
		**Mean**	**6.86**
		**%SD**	**0.16**

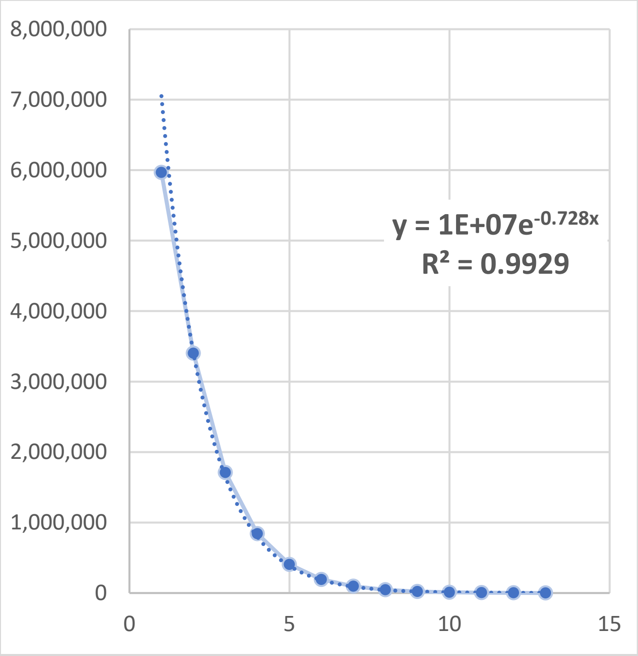

Test batch 2

Time (min)	CPM	Calculated decay constant (day^{-1})	Calculated half-life (day)
16/07/2024 17:00	2 941 633	-	-
22/07/2024 14:06	1 646 617	0.0986902142	7.02346414
29/07/2024 10:13	814 248	0.1009981527	6.862968894
05/08/2024 09:21	394 793	0.1020455246	6.792528957
12/08/2024 10:13	191 917	0.1021667709	6.78446793
19/08/2024 15:18	89 392	0.1029709003	6.731486067
26/08/2024 08:50	44 510	0.1030752045	6.724674316
02/09/2024 09:46	21 298	0.1033170218	6.708934967
09/09/2024 18:54	9 912	0.1033600690	6.706140843
16/09/2024 15:31	4 862	0.1034141315	6.70263503
24/09/2024 17:26	1 915	0.1047868665	6.614828785
01/10/2024 17:48	1 071	0.1027884832	6.743432329
07/10/2024 13:59	558	0.1034114645	6.702807894
		**Mean**	**6.76**
		**%SD**	**0.10**

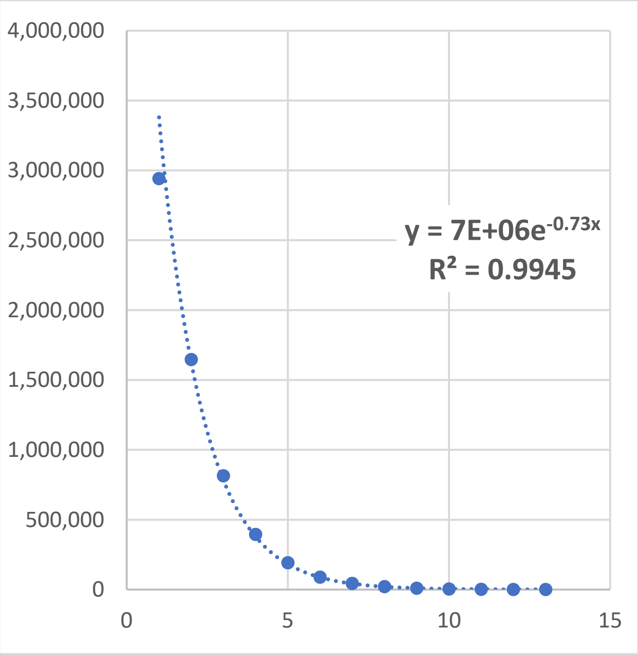

Test batch 3

Time (min)	CPM	Calculated decay constant (day-1)	Calculated half-life (day)
16/07/2024 17:00	8 281 025	-	-
22/07/2024 14:06	4 904 673	0.0890866164	7.780598354
29/07/2024 10:13	2 511 822	0.0938036570	7.389340699
05/08/2024 09:21	1 226 578	0.0970346020	7.143299053
12/08/2024 10:13	595 973	0.0984939010	7.037462966
19/08/2024 15:18	281 158	0.0997027800	6.95213494
26/08/2024 08:50	139 964	0.1003533572	6.907065194
02/09/2024 09:46	66 818	0.1010453242	6.859765022
09/09/2024 18:54	31 026	0.1014341545	6.833469297
16/09/2024 15:31	15 039	0.1018933368	6.802674269
24/09/2024 17:26	6 282	0.1026021689	6.755677659
01/10/2024 17:48	3 119	0.1023481827	6.772442483
07/10/2024 13:59	1 687	0.1025505638	6.759077225
		**Mean**	**7.00**
		**%SD**	**0.31**

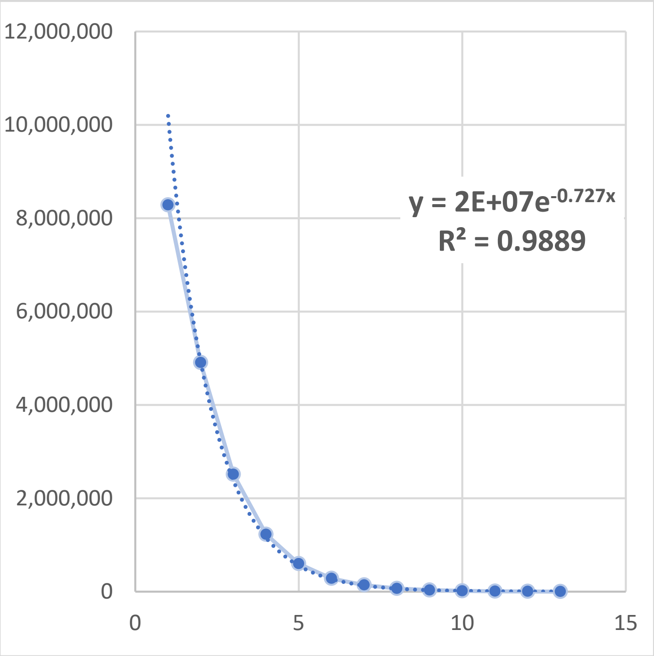

SVII – Radionuclide purity estimation of the [^{177}Lu]Lu-PSMA-ALB-56 test batches

Test batch 1

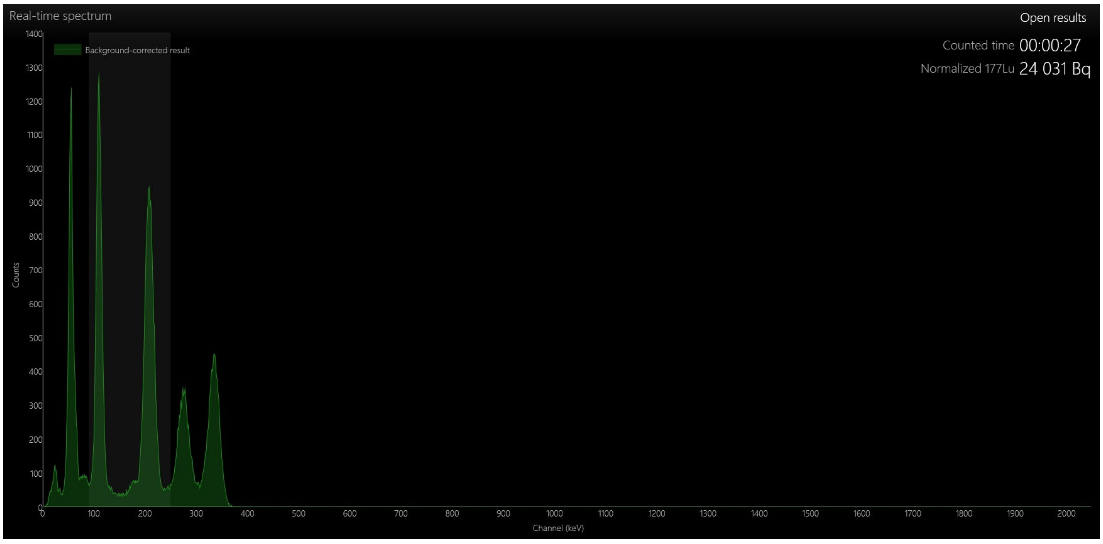

Test batch 2

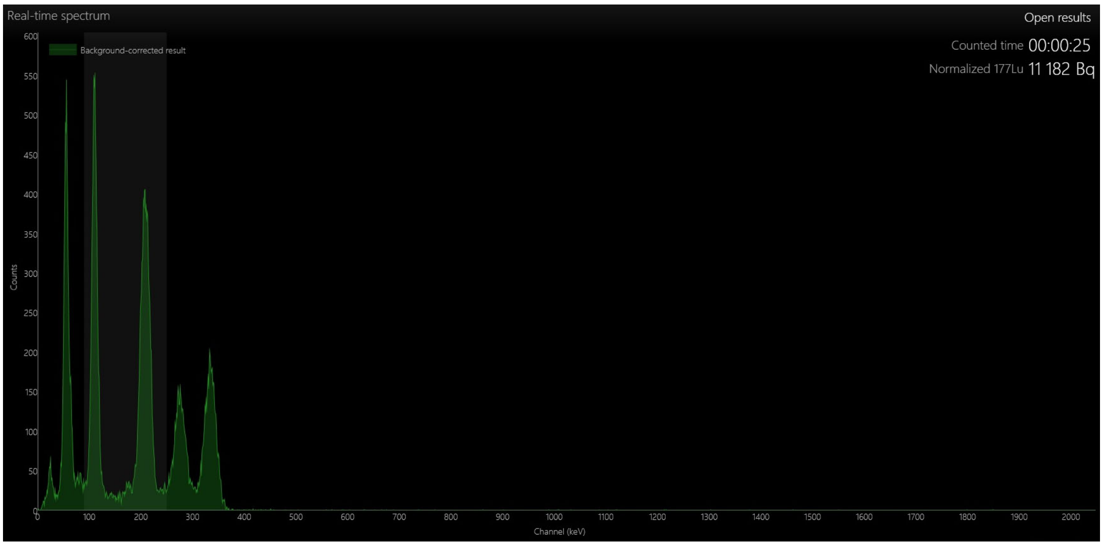

Test batch 3

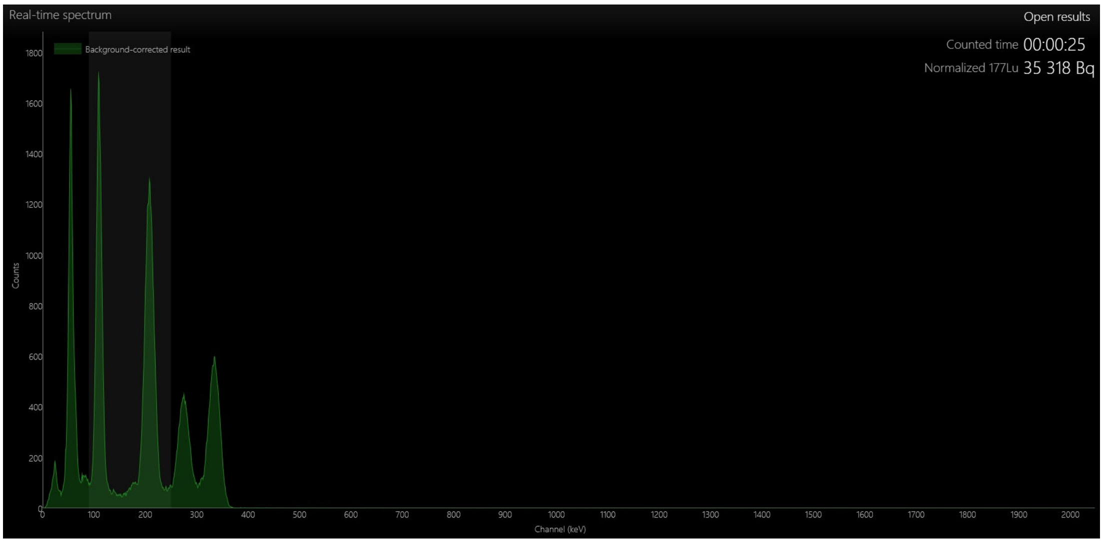

Supplement: Supplementary file 1 [file ijms-26-09642-s001.zip › ijms-3895750-supplementary.pdf]
